# Association of Diabetes Mellitus With Postoperative Complications and Mortality After Non-Cardiac Surgery: A Meta-Analysis and Systematic Review

**DOI:** 10.3389/fendo.2022.841256

**Published:** 2022-05-26

**Authors:** Xiaoying Zhang, Aisheng Hou, Jiangbei Cao, Yanhong Liu, Jingsheng Lou, Hao Li, Yulong Ma, Yuxiang Song, Weidong Mi, Jing Liu

**Affiliations:** Department of Anesthesiology, The First Medical Center of Chinese PLA General Hospital, Beijing, China

**Keywords:** diabetes mellitus, non-cardiac surgery, risk factor, postoperative complication, meta-analysis

## Abstract

**Background:**

Although a variety of data showing that diabetes mellitus (DM) (Type 1 or Type 2) is associated with postoperative complication, there is still a lack of detailed studies that go through the specific diabetic subgroups. The goal of this meta-analysis is to assess the relationship between DM and various complications after non-cardiac surgery.

**Methods:**

We searched articles published in three mainstream electronic databases (PubMed, EMBASE, Web of science) before November, 2020. A random effects model was conducted since heterogeneity always exist when comparing results between different types of surgery.

**Results:**

This paper included 125 studies with a total sample size of 3,208,776 participants. DM was a risk factor for any postoperative complication (Odds ratio (OR)=1.653 [1.487, 1.839]). The risk of insulin-dependent DM (OR=1.895 [1.331, 2.698]) was higher than that of non-insulin-dependent DM (OR=1.554 [1.061, 2.277]) for any postoperative complication. DM had a higher risk of infections (OR=1.537 [1.322, 1.787]), wound healing disorders (OR=2.010 [1.326, 3.046]), hematoma (OR=1.369 [1.120, 1.673]), renal insufficiency (OR=1.987 [1.311, 3.013]), myocardial infarction (OR=1.372 [0.574, 3.278]). Meanwhile, DM was a risk factor for postoperative reoperation (OR=1.568 [1.124, 2.188]), readmission (OR=1.404 [1.274, 1.548]) and death (OR=1.606 [1.178, 2.191]).

**Conclusions:**

DM is a risk factor for any postoperative complications, hospitalization and death after non-cardiac surgery. These findings underscore the importance of preoperative risk factor assessment of DM for the safe outcome of surgical patients.

## 1 Introduction

Each year more than 300 million surgeries are performed in the world (1). The baseline 30-day mortality of hospitalized patients undergoing non-cardiac surgery is 1.5% worldwide, primarily depending on surgical method, surgical decision-making or technique, and comorbidities (2). It is important to identify factors that increase the risk of surgery before making clinical decisions (3). The preoperative identification of risk factors has important clinical implications. First, it helps surgeons correct those risk factors that can be optimized prior to surgery to reduce surgical risk. Second, it directs patients to undergo low-risk surgery or transfer to appropriate medical institutions with stronger technical ability. Third, it is beneficial to make correct decisions based on risk-benefit evaluation. To date, it is still a very tough task to preoperatively identify high-risk patients or the subgroup population who would benefit most from surgery.

Diabetes mellitus (DM) (Type 1 or Type 2) is a multifaceted metabolic disease that affects more than 340 million people worldwide (4). They are at high risk for microvascular (neuropathy, nephropathy or retinopathy) or macrovascular (peripheral vascular, cardiovascular disease) complications, both of which increase perioperative morbidity and mortality (5). Surgical patients with DM are more likely to have prolonged hospital stays, admission to intensive care units, myocardial infarction, respiratory infections, poor wound healing, and increased risk of general morbidity and mortality (6–9). It is important for surgeons to be aware of possible complications and associated contributing factors so that they can be appropriately counseled preoperatively. Clinicians should develop direct strategies in the perioperative period to minimize surgical risks based on existing DM screening programs (10).

To date, there seems to lack detailed studies that go through this specific diabetic subgroup, although there are very convincing data showing that DM is associated with a variety of postoperative complications (5). After all, exactly which complications are associated with DM remains controversial. In order to provide clinicians with a reference to assess the surgical risk, we performed meta-analysis and systematic review of various complications after noncardiac surgery in patients with DM.

## 2 Methods

### 2.1 Protocol and Guidance

This meta-analysis followed the Preferred Reporting Items for Systematic Reviews and Meta analyses (PRISMA) guidelines (11). No registration details are available.

### 2.2 Eligibility Criteria

In this manuscript were included studies, which described DM as a preoperative risk factor. These studies that presented postoperative complications, mortality, morbidity, length of ICU stay and prolonged hospital stay, providing adjusted or unadjusted relative risk (RR) or odds ratio (OR) with 95% confidence interval (CI); or providing relevant information that can be used to figure out RR or OR. The studies’ design relied on retrospective data.

### 2.3 Information Sources and Search Strategy

We searched for articles published before November 30, 2020 regardless of language in a total of three electronic databases (PubMed, EMBASE, Web of science). We restricted our search to human studies. In the search we used the following terms: “diabetes”, “postoperative complications”, “surgical procedures”, “operative,” “hospitalization”, “risk factors”, “treatment outcome”, “perioperative care”, “perioperative period”, “reoperation” and “wound healing”. References of identified studies, recent guidelines and reviews on this topic were also selected by manual screening. Studies on cardiac surgery were excluded.

### 2.4 Study Selection and Data Collection

Two authors independently selected studies by screening titles and abstracts, and any disagreements were resolved by the senior author. Data extraction was performed independently by two authors. Study characteristics including author, publication year, country, sample size, mean or median age, number of patients with DM, type of DM, and type of surgery were extracted. Data were extracted for pooling, including total number of subjects, number of events of various complications, RR or OR. If the data were only available as graphs, the free software Plot Digitizer was used to estimate from the graphs. Quality assessment was using the Newcastle-Ottawa scale (NOS) for assessing quality of observational studies.

### 2.5 Definition of Outcomes

Our outcome measure is the OR of the incidence of complications in diabetic versus nondiabetic patients after surgery. It also shows the OR of mortality, readmission, reoperation, and prolonged length of stay (LOS). We have pooled OR for 7 postoperative complications, including any complication, infections, wound healing disorders (WHD), venous thromboembolism (VTE), hematoma, renal insufficiency, and myocardial infarction (MI).

### 2.6 Statistical Analysis

Homogeneity of effect estimates was tested using the Cochran Q and I² statistics (12, 13). A random effects model was conducted because of heterogeneity always exist when comparing results between different types of surgery, and subgroup analyses were performed. All outcomes were presented as OR with 95% CI. All analyses were performed using Stata/SE version 15.0 (StataCorp, College Station, TX, USA). Publication bias was assessed by evaluating small‐study effects with comparison adjusted funnel plot symmetry if 10 or more studies were available.

## 3 Results

### 3.1 Study Selection and Study Characteristics

**Figure 1:**
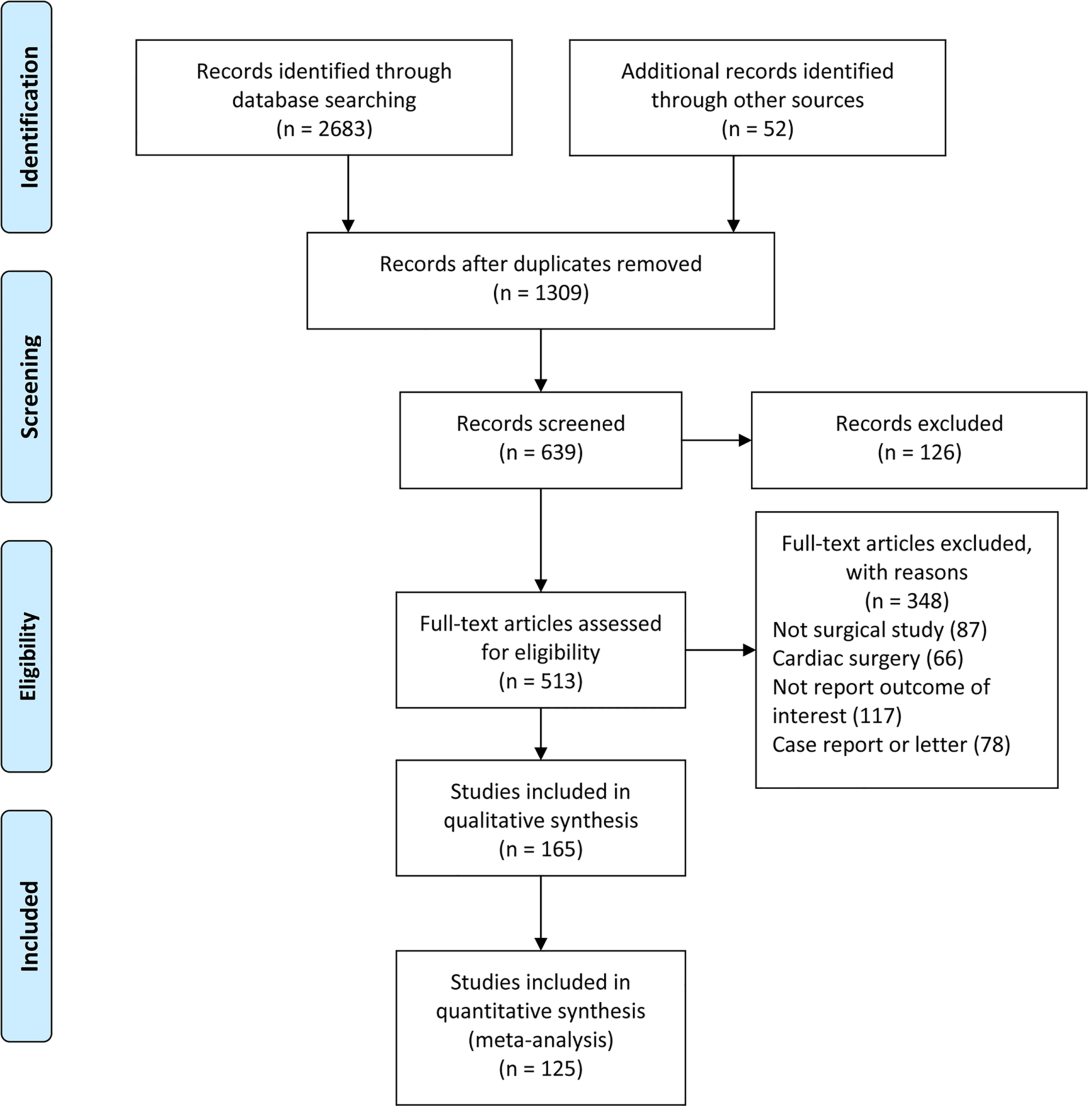
Flowchart of study selection.

Our literature search yielded a total of 2,737 retrievals, 125 studies were used for meta-analysis. **Figure 1**. A total of 72 studies were from the United States, accounting for more than half of the 125 studies included. The number of patients with DM was 356,300, accounting for 11.1% of the huge sample size of 3,208,776. The vast majority of studies focused on all types of DM, and only eight studies distinguished between IDDM and NIDDM. The types of surgery mainly covered orthopedic surgery, oncological surgery, transplantation surgery, plastic surgery, weight loss surgery, oral surgery, neurological surgery, ophthalmological surgery, etc., with the exception of cardiac surgery. The quality of the included studies was assessed using the NOS criteria. The NOS quality scores of the included studies ranged from 6 to 9 points (**Table 1**).

**Table 1:** Characteristics of included studies.

Study	Year	Age	Sample size	Number of DM	Country	Surgery		NOS Score	
							Selection	Comparability	Outcome
Afshari et al. (14)	2016	–	1493	166	USA	thighplasty	☆☆☆☆	☆☆	☆☆☆
Aigner et al. (15)	2017	72.5(6.1)	237	26	Germany	open reduction and internal fixation of geriatric ankle fractures	☆☆☆☆	☆☆	☆☆☆
Akhter et al. (16)	2016	–	1196	133	India	surgery	☆☆☆☆	☆☆	☆☆☆
Ammori et al. (17)	2018	69	6985	1389	USA	gastrectomy for malignancy	☆☆☆	☆☆	☆☆☆
Arnold et al. (18)	2014	60.1 (10.7)	278	42	USA	surgical decompression, in cervical spondylotic myelopathy	☆☆☆☆	☆☆	☆☆☆
Bailey et al. (19)	2003	63.4(9.9)	1777	221	USA	esophagectomy	☆☆☆	☆☆	☆☆☆
Bailón-Cuadrado et al. (20)	2019	68.6(11.1)	180	26	Spain	curative surgery for colorectal cancer	☆☆☆	☆☆	☆☆☆
Bamba et al. (21)	2016	40.9(13.9)	129007	2368	USA	aesthetic surgery	☆☆☆☆	☆	☆☆
Bascom et al. (22)	2016	43.9	829	43	Canada	bulbar urethroplasty	☆☆☆	☆☆	☆☆☆
Belmont et al. (23)	2015	67.3(10.2)	15321	2795	USA	total knee arthroplasty	☆☆☆☆	☆☆	☆☆☆
Belmont et al. (24)	2014	50.3 (18.2)	3328	426	USA	ankle fracture fixation	☆☆☆☆	☆☆	☆☆☆
Benrashid et al. (25)	2020	64.7	504	152	USA	vascular procedures requiring infrainguinal incisions	☆☆☆	☆☆	☆☆☆
Bohl et al. (2)	2019	–	7582	842	USA	open reduction and internal fixation of closed ankle fractures	☆☆☆☆	☆☆	☆☆☆
Bolognesi et al. (26)	2008	61.0-76.0	751340	64262	USA	total hip and total knee arthroplasty	☆☆☆☆	☆	☆☆
Bower et al. (27)	2010	61.6(14.1)	1343	329	Hong Kong	surgical outcomes of noncardiovascular patients	☆☆☆☆	☆	☆☆☆
Browne et al. (28)	2007	48.9(18.16)	197461	11135	USA	lumbar fusion	☆☆☆☆	☆	☆☆
Bruggeman et al. (29)	2004	43	167	19	USA	open achilles tendon repair	☆☆☆☆	☆☆	☆☆☆
Buchanan et al. (30)	2018	>18	93920	10425	USA	non-emergent craniotomy	☆☆☆☆	☆☆	☆☆☆
Bur et al. (31)	2016	64.2	7605	844	USA	head and neck cancer surgery	☆☆☆	☆☆	☆☆☆
Cammarata et al. (32)	2019	–	7030	770	USA	abdominal panniculectomy	☆☆☆	☆☆	☆☆☆
Chen et al. (33)	2009	–	195	30	USA	spinal arthrodesis	☆☆☆☆	☆☆	☆☆☆
Chen et al. (34)	2019	53.9(12.4)	207	23	China	open hepatectomy	☆☆☆	☆☆	☆☆☆
Chiu et al. (35)	2020	54.6(11.5)	40	4	Taiwan	sequential free flap reconstruction	☆☆	☆☆	☆☆
Ciufo et al. (36)	2019	64.5 (13.3)	4631	3233	USA	below knee amputation	☆☆☆☆	☆☆	☆☆☆
Cook et al. (37)	2008	53(13.20)	37732	3432	USA	cervical fusion	☆☆☆☆	☆☆	☆☆
Cote et al. (38)	2019	–	1005	112	USA	microvascular decompression	☆☆☆	☆☆	☆☆☆
Courtney et al. (39)	2017	65.9	169406	25913	USA	total joint arthroplasty	☆☆☆☆	☆	☆☆
Cutler et al. (40)	2020	54-81	414	29	USA	total elbow arthroplasty	☆☆☆☆	☆☆	☆☆☆
Dodd et al. (41)	2016	53.4(18.4)	6800	836	USA	ankle fractures	☆☆☆☆	☆☆	☆☆☆
Duque et al. (42)	1997	–	605	46	Spain	thoracotomy for bronchogenic carcinoma	☆☆☆	☆☆	☆☆☆
Farivar et al. (43)	2017	73 (9)	5881	945	USA	endovascular aneurysm repair of infrarenal abdominal aortic aneurysms	☆☆☆	☆☆	☆☆☆
Fischer et al. (44)	2014	58	47443	7288	USA	mastectomy alone compared to immediate breast reconstruction	☆☆☆	☆☆	☆☆☆
Franck et al. (45)	2018	55.2	60	7	USA	local muscle flap closure following spinal tumor extirpation	☆☆☆	☆☆	☆☆☆
Freire et al. (46)	2015	47 (12–79)	819	150	Brazil	kidney transplantation	☆☆☆	☆☆	☆☆☆
Fu et al. (47)	2016	–	3671	455	USA	anterior cervical discectomy and fusion	☆☆☆☆	☆☆	☆☆☆
Ganesh et al. (48)	2005	62.9(13.8)	160598	9174	USA	ankle fracture	☆☆☆☆	☆	☆☆
Golinvaux et al. (49)	2014	–	15480	2437	USA	elective lumbar fusion	☆☆☆☆	☆☆	☆☆☆
Gupta et al. (50)	2017	40.2(13.9)	183914	20414	USA	aesthetic surgical	☆☆☆☆	☆	☆☆
Gupta et al. (51)	2016	40.9(13.9)	127961	2346	USA	aesthetic surgery	☆☆☆☆	☆	☆☆
Gupta et al. (52)	2016	59.24 (9.4)	11300	303	USA	facelift	☆☆☆☆	☆	☆☆☆
Gupta et al. (53)	2017	40.9 (13.9)	129007	2368	USA	aesthetic breast surgery	☆☆☆☆	☆	☆☆
Hadaya et al. (54)	2020	60.8 (12.6)	22739	2524	USA	elective pneumonectomy	☆☆☆	☆☆	☆☆☆
Hardt et al. (55)	2017	64.4(11.9)	370	37	Germany	elective rectal cancer resection	☆☆☆	☆☆	☆☆☆
Hunecke et al. (56)	2019	43.7(12.7)	121	18	Germany	abdominoplasty after massive weight loss	☆☆☆☆	☆	☆☆☆
Inabnet et al. (57)	2010	44.22	3802	1323	USA	non-lap band primary and revisional bariatric surgical procedures	☆☆☆☆	☆	☆☆☆
Janczak et al. (58)	2019	67.9(6.7)	205	46	Poland	elective open surgery for infrarenal aortic aneurysms	☆☆☆	☆☆	☆☆☆
John and Thuluvath (59)	2001	53.6(6.7)	171	57	USA	liver transplantation	☆☆☆	☆☆	☆☆☆
Kantar et al. (60)	2018	54.4(11.0)	7035	770	USA	abdominal panniculectomy	☆☆☆☆	☆	☆☆☆
Karthikesalingam et al. (61)	2011	40 (21–70)	123	14	UK	abdominoplasty	☆☆☆	☆☆	☆☆☆
Kauvar et al. (62)	2017	77 (9)	3344	648	USA	elective endovascular aortic aneurysm repair	☆☆☆	☆☆	☆☆☆
Koch et al. (63)	2015	53 (15)	405	79	Germany	kidney transplantation	☆☆☆	☆☆	☆☆☆
Lange et al. (64)	2009	72 (50- 84)	121	27	Netherlands	peripheral vascular surgery	☆☆☆	☆☆	☆☆☆
Lee et al. (65)	2018	–	2301	421	Korea	elective posterior lumbar fusion	☆☆☆☆	☆☆	☆☆☆
Lewin et al. (66)	2014	39.6 (13.8)	512	14	Sweden	breast reduction surgery	☆☆☆☆	☆	☆☆☆
Li et al. (67)	2017	–	3024	223	China	gastric cancer	☆☆☆	☆☆	☆☆☆
Lindqvist et al. (68)	2019	57.4 (18-91)	886	22	Sweden	sentinel lymph node biopsy for cutaneous melanoma	☆☆☆	☆☆	☆☆☆
Lopez Ramos et al. (69)	2018	61	40802	4880	USA	cranial neurosurgery	☆☆☆	☆☆	☆☆☆
Louie et al. (70)	2017	51.3	3251	387	USA	open reduction internal fixation of ankle fractures	☆☆☆☆	☆☆	☆☆☆
Lv et al. (71)	2015	49.7(8.8)	438	140	China	liver transplantation	☆☆☆	☆☆	☆☆☆
Ma et al. (72)	2019	62.6(10.5)	545	61	China	radical gastrectomy	☆☆☆	☆☆	☆☆☆
Maradit Kremers et al. (73)	2015	66.2(12.6)	20171	3507	USA	total hip and knee arthroplasty	☆☆☆☆	☆☆	☆☆
Matsuda et al. (74)	2009	66.2(8.8)	80	9	Japan	abdominoperineal resection	☆☆☆	☆☆	☆☆☆
McElvany et al. (75)	2019	69.5(9.7)	8819	1874	USA	shoulder arthroplasty	☆☆☆☆	☆☆	☆☆☆
Meding et al. (76)	2003	–	5220	329	USA	total knee replacement	☆☆☆☆	☆☆	☆☆☆
Menenakos et al. (77)	2010	37	261	36	Greece	laparoscopic sleeve gastrectomy	☆☆☆	☆☆	☆☆☆
Michalak et al. (78)	2016	53.3(13.5)	1141	115	USA	cerebrovascular surgery	☆☆☆	☆☆	☆☆☆
Moon et al. (79)	2008	67.6 (50–86)	342	171	Korea	total knee arthroplasty	☆☆☆☆	☆☆	☆☆☆
Moon et al. (80)	2018	49.9(11.5)	5538	615	USA	sleeve gastrectomy	☆☆☆	☆☆	☆☆☆
Morgan et al. (81)	2015	48	12062	1339	Australia	bariatric surger	☆☆☆☆	☆	☆☆☆
Nair et al. (82)	2009	52 (19)	221	55	USA	liver transplantation	☆☆☆	☆☆	☆☆☆
Newman et al. (83)	2014	60.4(12.9)	3352	406	USA	total knee and total hip arthroplasty	☆☆☆☆	☆☆	☆☆☆
Nguyen et al. (84)	2019	62(11.9)	563	69	Canada	gynecologic oncology	☆☆☆	☆☆	☆☆☆
Nguyen et al. (85)	2016	48.65(12.72)	2294	126	USA	brachioplasty	☆☆☆☆	☆☆	☆☆☆
Okamura et al. (86)	2017	63 (8)	300	35	Japan	esophagectomy	☆☆☆	☆☆	☆☆☆
Palmerola et al. (87)	2016	64 (54-94)	191	21	USA	urologic surgery	☆☆☆	☆☆	☆☆☆
Park et al. (88)	2016	51.44(10.8)	7948	1284	Korea	anterior cervical discectomy and fusion for cervical spondylotic, radiculopathy and myelopathy	☆☆☆☆	☆☆	☆☆☆
Patton et al. (89)	2015	55.4	87	6	USA	total ankle arthroplast	☆☆☆☆	☆☆	☆☆☆
Pearse et al. (90)	2012	56·7 (18·5)	46539	5576	UK	non-cardiac surgery	☆☆☆☆	☆	☆☆
Plano et al. (91)	2019	57.7 (27-86)	303	34	Spain	unplanned surgery in cervical spondylotic myelopathy surgically treated	☆☆☆☆	☆☆	☆☆☆
Ponce et al. (92)	2014	69 (13)	66485	13730	USA	shoulder arthroplasty	☆☆☆☆	☆	☆☆
Pugely et al. (93)	2013	52.6 (16.1)	4310	455	USA	lumbar discectomy	☆☆☆☆	☆☆	☆☆☆
Qin et al. (94)	2014	55.9 (10.2)	29736	1478	USA	breast reconstruction	☆☆☆☆	☆☆	☆☆
Raikin et al. (95)	2010	–	106	11	USA	total ankle arthroplasty	☆☆☆☆	☆☆	☆☆☆
Rao et al. (96)	2020	69.9(8.4)	1074	433	USA	shoulder arthroplasty	☆☆☆☆	☆☆	☆☆☆
Rensing et al. (97)	2017	44(13.3)	1626	79	USA	primary repair of achilles tendon ruptures	☆☆☆☆	☆☆	☆☆☆
Roche et al. (98)	2018	61.24 (12.8)	9439	1402	USA	parathyroidectomy for primary hyperparathyroidism	☆☆☆☆	☆☆	☆☆☆
Rubel et al. (99)	2019	57.5(16.2)	169788	31289	USA	elective primary lumbar spine surgery	☆☆☆☆	☆	☆☆
Sakai et al. (100)	2011	–	107	12	Japan	surgery for laryngeal and hypopharyngeal cancers	☆☆☆	☆☆	☆☆☆
Sanni et al. (101)	2014	44.0 (12.1)	20308	5268	USA	bariatric surgery	☆☆☆☆	☆	☆☆
Schemitsch et al. (102)	2015	34.9	153	6	Canada	plate fixation of the midshaft clavicle	☆☆☆☆	☆☆	☆☆☆
Schimmel et al. (103)	2010	51 (16.8)	171	8	Netherlands	spinal fusion	☆☆☆☆	☆☆	☆☆☆
Schipper et al. (104)	2015	65.7(10.1)	12122	2394	USA	ankle arthrodesis and total ankle arthroplasty	☆☆☆☆	☆☆	☆☆☆
Schlottmann et al. (105)	2017	63 (10.3)	4053	229	USA	esophagectomy	☆☆☆	☆☆	☆☆☆
Schürner et al. (106)	2018	40 (32–49)	711	200	Switzerland	primary roux-en-y gastric bypass surgery	☆☆☆	☆☆	☆☆☆
Shah et al. (107)	2019	65 (11)	3344	346	USA	thumb cmc joint arthroplasty	☆☆☆☆	☆☆	☆☆☆
Shigeishi et al. (108)	2015	41(5-84)	324	12	Japan	oral surgery	☆☆☆	☆☆	☆☆☆
Shimada et al. (109)	1994	57.5	209	23	Japan.	hepatic resection	☆☆☆	☆☆	☆☆☆
Smith et al. (110)	2017	45.78(17.70)	272	30	USA	tibia fractures treated with intramedullary fixation	☆☆☆☆	☆☆	☆☆☆
Söderbäck et al. (111)	2019	71.1 (11.6)	30050	952	Sweden	colorectal cancer surgery	☆☆☆	☆☆	☆☆☆
Sood et al. (112)	2015	62 (54–71)	3820	755	USA	nephrectomy	☆☆☆	☆☆	☆☆☆
Sood et al. (113)	2017	69 (61-76)	1118	214	USA	radical cystectomy	☆☆☆	☆☆	☆☆☆
Sørensen et al. (114)	2002	64	425	47	Denmark	breast cancer surgery	☆☆☆	☆☆	☆☆☆
Souza et al. (115)	2007	45.6(10.4)	55	4	Brazil	liver transplantations	☆☆☆	☆	☆☆
Spinazzi et al. (116)	2015	55.9(15.2)	15317	2493	USA	pituitary surgery	☆☆☆	☆☆	☆☆☆
Stein et al. (117)	2011	–	221594	64569	USA	cataract surgery	☆☆☆☆	☆	☆☆
Suda et al. (118)	2016	57.2	108	25	Germany	arthrodesis	☆☆☆☆	☆☆	☆☆☆
Takao et al. (119)	2008	> 80	255	68	Japan	urological surgery	☆☆☆	☆☆	☆☆☆
Tang et al. (120)	2014	66.8(5.5)	236	74	China	spinal fusion and instrumentation	☆☆☆☆	☆☆	☆☆☆
Terho et al. (121)	2016	63 (20–94)	373	68	Finland	laparoscopic cholecystectomy	☆☆☆	☆☆	☆☆☆
Tetreault et al. (122)	2016	56.4 (11.9)	479	59	Canada	surgery for the treatment of cervical spondylotic myelopathy	☆☆☆☆	☆☆	☆☆☆
Timmermans et al. (123)	2018	49.1(9.2)	97	5	Netherlands	free diep flap breast reconstructions	☆☆☆☆	☆☆	☆☆☆
Toboni et al. (124)	2018	60.4	4260	540	USA	ovarian cancer	☆☆☆	☆☆	☆☆☆
Tokgöz et al. (125)	2011	61.6(12.1)	47	8	Turkey	radical nephrectomy	☆☆	☆☆	☆☆☆
Venara et al. (126)	2014	74 (18-109)	166	25	France	treatment of incarcerated hernias, especially in case of bowel resection	☆☆☆	☆☆	☆☆☆
Wadhwa et al. (127)	2017	54.2(16.7)	9853	1690	USA	surgery for lumbar degenerative disease	☆☆☆☆	☆☆	☆☆☆
Wang et al. (128)	2020	72 (65-86)	118	7	China	radial forearm-free flap	☆☆☆☆	☆☆	☆☆☆
Wang et al. (129)	2017	–	1657	184	China	laparoscopy-assisted total gastrectomy	☆☆☆	☆☆	☆☆☆
Webb et al. (130)	2017	–	114102	20248	USA	total knee arthroplasty	☆☆☆☆	☆	☆☆
Weir et al. (131)	2019	52.2 (14.7)	5222	580	UK	lumbar spinal surgery	☆☆☆☆	☆☆	☆☆☆
Winocour et al. (132)	2017	45.5 (10.3)	129007	2368	USA	cosmetic surgery	☆☆☆☆	☆	☆☆
Wukich et al. (133)	2010	46.7	1000	190	USA	foot and ankle surgery	☆☆☆☆	☆☆	☆☆☆
Yamauchi et al. (134)	2013	–	1438	148	Japan	lung cancer operations	☆☆☆	☆☆	☆☆☆
Zanaty et al. (135)	2015	46.5(12.7)	348	52	USA	cranioplasty	☆☆☆	☆☆	☆☆☆
Zhang et al. (136)	2015	65.8(11.3)	119	39	China	pancreatoduodenectomy	☆☆☆	☆☆	☆☆☆
Zhou et al. (137)	2016	65.9(12.0)	2795	228	China	gastrectomy for gastric cancer	☆☆☆	☆☆	☆☆☆
Total studies 125			3208776	356300					

DM, Diabetes mellitus; NOS, Newcastle-Ottawa scale.

### 3.2 Synthesis of Results

#### 3.2.1 Any Complication

**Figure 2:**
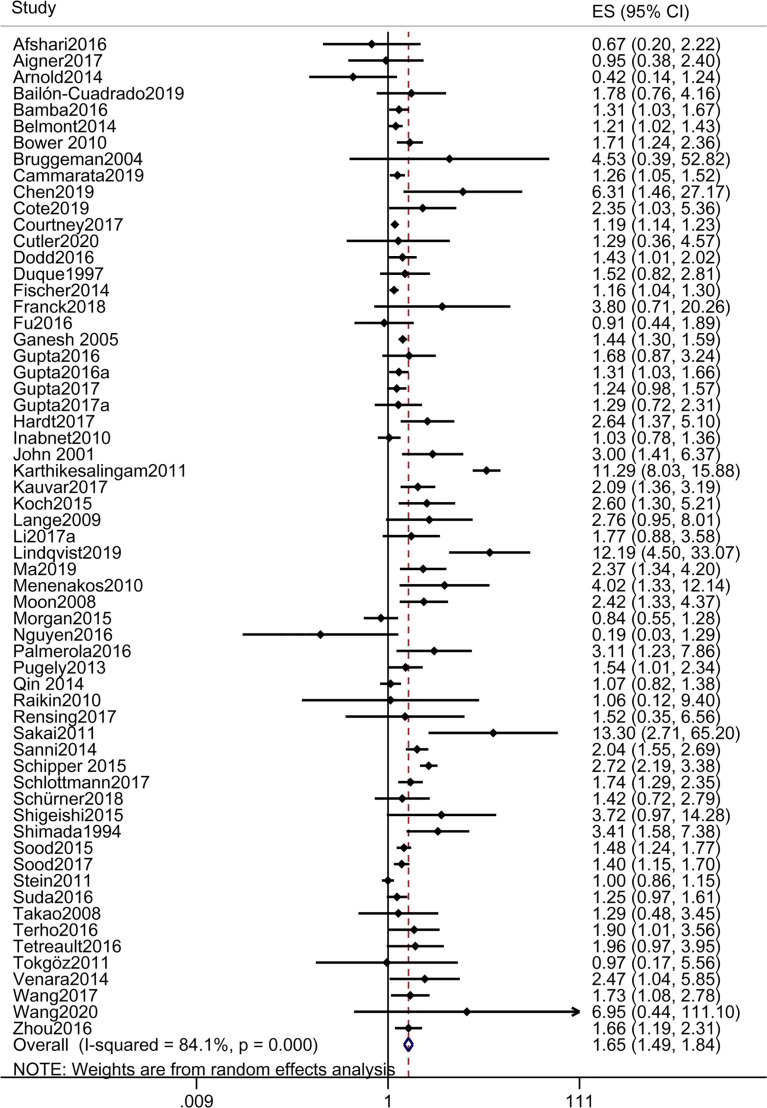
Forest plot of odds ratio of any postoperative complication in patients with DM vs those without DM.

A total of 61 studies reported any complication. The pooled OR of any complication in patients with DM vs those without DM was 1.653 [1.487, 1.839], suggesting that DM was a risk factor for any postoperative complication (**Table 2** and **Figure 2**). The results of subgroup analyses showed that OR of any complication in patients with IDDM and NIDDM vs those without DM were 1.895 [1.331, 2.698] and 1.554 [1.061, 2.277], respectively, suggesting that IDDM and NIDDM were risk factors for any postoperative complication, and the risk of IDDM was higher than that of NIDDM (**Table 2** and **Supplementary Figures S1, S2**).

**Table 2:** Outcomes.

Complications	Odds ratio (95%CI)	Studies included
Any complications	1.653 (1.487, 1.839)	61
Any complications-IDDM	1.895 (1.331, 2.698)	8
Any complications-NIDDM	1.554 (1.061, 2.277)	7
Infections	1.537 (1.322, 1.787)	40
VTE	1.189 (0.759, 1.864)	11
Wound healing disorders	2.010 (1.326, 3.046)	9
Hematoma	1.369 (1.120, 1.673)	8
Renal insufficiency/failure	1.987 (1.311, 3.013)	5
MI	1.372 (0.574, 3.278)	4
Length of stay	1.581 (1.271, 1.968)	7
Readmission	1.404 (1.274, 1.548)	15
Reoperation	1.568 (1.124, 2.188)	11
Mortality	1.606 (1.178, 2.191)	18
Mortality-Cancer surgery	1.052 (0.419,2.643)	3
Mortality-Orthopedic surgery	1.817 (1.136,2.906)	8
Mortality-Hemangioma resection	1.509 (0.889,2.561)	3
Mortality-transplant	1.214 (0.410,3.592)	2

IDDM, Insulin-Dependent Diabetes Mellitus; MI, Myocardial infarction; NIDDM, Non-Insulin-Dependent Diabetes Mellitus; VTE, Venous Thromboembolism.

#### 3.2.2 Organ or System Complications

##### 3.2.2.1 Infections

A total of 40 studies reported infections. The pooled OR of infections in patients with DM vs those without DM was 1.537 [1.322, 1.787], suggesting that DM was a risk factor for postoperative infections (**Table 2** and **Supplementary Figure S3**).

##### 3.2.2.2 Venous Thromboembolism

A total of 11 studies reported VTE. The pooled OR of VTE in patients with DM vs those without DM was 1.189 [0.759, 1.864], which was statistically insignificant. This result suggested that DM was not a risk factor for postoperative VTE (**Table 2** and **Supplementary Figure S4**).

##### 3.2.2.3 Wound Healing Disorders

A total of nine studies reported WHD. The pooled OR of WHD in patients with DM vs those without DM was 2.010 [1.326, 3.046], suggesting that DM was a risk factor for postoperative WHD (**Table 2** and **Supplementary Figure S5**).

##### 3.2.2.4 Hematoma

A total of eight studies reported hematoma. The pooled OR of hematoma in patients with DM vs those without DM was 1.369 [1.120, 1.673], suggesting that DM was a risk factor for postoperative hematoma (**Table 2** and **Supplementary Figure S6**).

##### 3.2.2.5 Renal Insufficiency

A total of five studies reported renal insufficiency. The pooled OR of renal insufficiency in patients with DM vs those without DM was 1.987 [1.311, 3.013], suggesting that DM was a risk factor for postoperative renal insufficiency (**Table 2** and **Supplementary Figure S7**).

##### 3.2.2.6 Myocardial Infarction

A total of four studies reported MI. The pooled OR of MI in patients with DM vs those without DM was 1.372 [0.574, 3.278], which was statistically insignificant. This result suggested that DM was not a risk factor for postoperative MI (**Table 2** and **Supplementary Figure S8**).

#### 3.2.3 Hospitalization

A total of seven studies reported LOS. The pooled OR of LOS in patients with DM vs those without DM was 1.987 [1.311, 3.013], suggesting that DM was a risk factor for extended LOS after surgery. A total of eleven and fifteen studies reported reoperation and readmission with the pooled OR 1.568 [1.124, 2.188] and 1.404 [1.274, 1.548], respectively. The results suggested that DM was a risk factor for postoperative reoperation and readmission (**Table 2** and **Supplementary Figures S9–S11**).

#### 3.2.4 Survival

**Figure 3:**
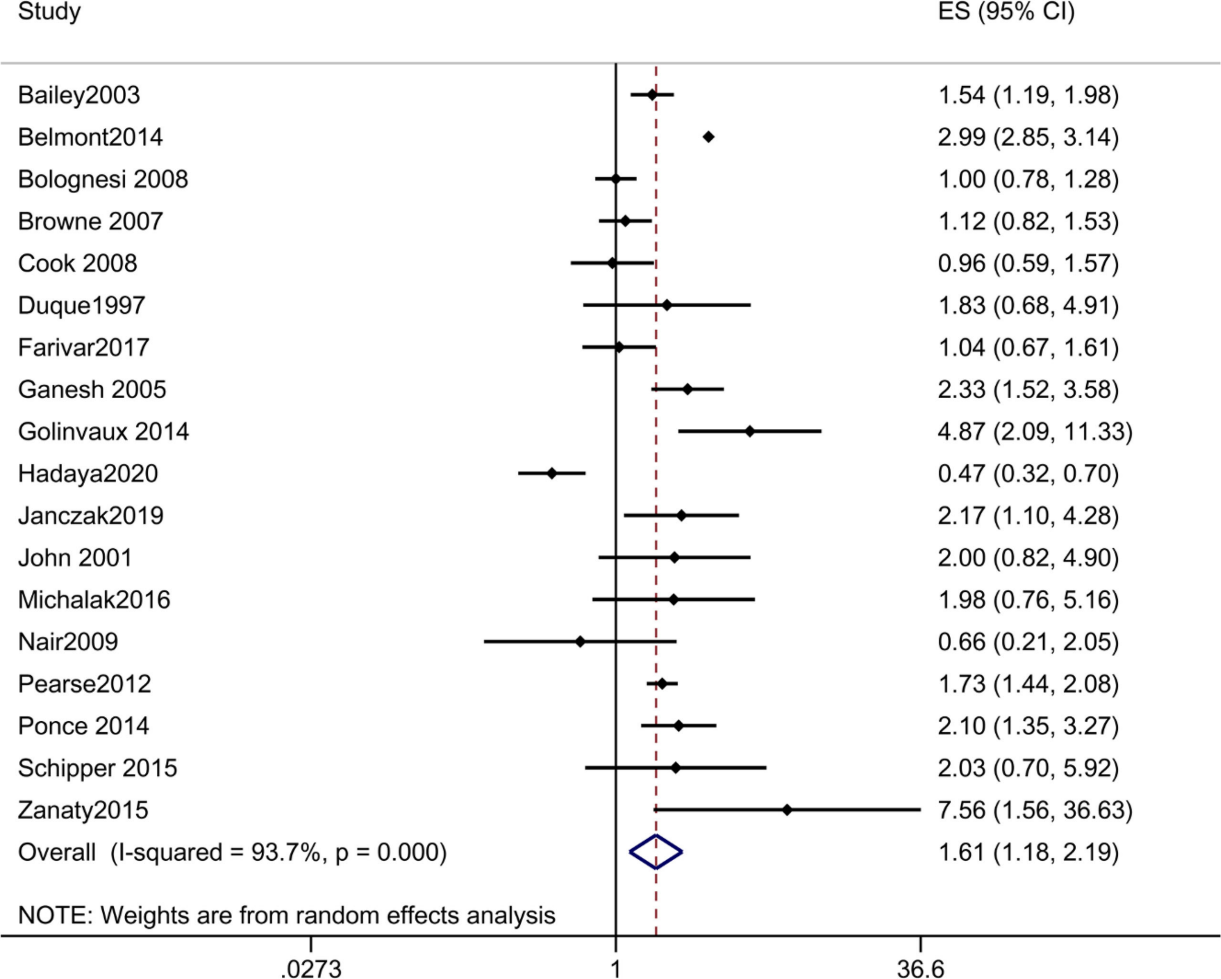
Forest plot of odds ratio of postoperative mortality in patients with DM vs those without DM.

A total of 18 studies reported mortality. The pooled OR of mortality in patients with DM vs those without DM was 1.606 [1.178, 2.191], suggesting that DM was a risk factor for postoperative death (**Figure 3**). The results of subgroup analyses revealed that the pooled OR of mortality in patients with DM vs those without DM was 1.817 [1.136, 2.906] after orthopedic surgery, while the pooled OR of mortality were 1.052 [0.419, 2.643], 1.509 [0.889, 2.561] and 1.214 [0.410, 3.592] after cancer surgery, hemangioma resection and transplant, respectively. The results suggested that DM was a risk factor for death after orthopedic surgery, not for death after cancer surgery, hemangioma resection and transplant (**Table 2** and **Supplementary Figures S12–S15**).

### 3.3 Subgroup Analysis

According to the type of surgery, we had three subgroups: general surgery, orthopedics and aesthetic surgery. Only the results of general surgery are slightly different from the overall results, the pooled OR of VTE in patients with DM vs those without DM was 3.627[2.405, 5.469], suggesting that DM was a risk factor for postoperative VTE. The analysis results of the other two subgroups were consistent with the overall results. (**Table 3** and **Supplementary Figures S16–32**).

**Table 3:** Outcomes of subgroup analysis.

Aesthetic Surgery	OR (95%CI)	Studies included
Complications		
Any complications	1.582 (1.044, 2.396)	10
Infections	1.670 (1.344, 2.074)	7
VTE	0.270 (0.069, 1.059)	2
Hematoma	1.362 (1.074, 1.727)	5
**General Surgery**		
Complications	OR (95%CI)	**Studies included**
Any complications	1.847 (1.595, 2.139)	32
Infections	1.732 (1.268, 2.364)	14
VTE	3.627 (2.405, 5.469)	2
Wound healing disorders	2.053 (1.126, 3.740)	5
Renal insufficiency/failure	2.259 (1.234, 4.135)	4
Mortality	1.400 (0.976, 2.010)	10
**Orthopedic Surgery**		
Complications	OR (95%CI)	**Studies included**
Any complications	1.409 (1.194, 1.664)	19
Infections	1.425 (1.136, 1.786)	19
VTE	0.975 (0.789, 1.206)	7
Wound healing disorders	2.355 (1.380, 4.017)	3
Hematoma	1.607 (0.821, 3.145)	3
MI	1.372 (0.574, 3.278)	4
Mortality	1.817 (1.136, 2.906)	8

MI, Myocardial infarction; VTE, Venous Thromboembolism.

### 3.4 Publication Bias

We performed Egger’s test based on six comparisons (any complication, infection, VTE, readmission, reoperation, and mortality) with more than ten included studies. *P*-value of Egger’s test for VTE was 0.688, suggesting no publication bias, while *P*-values for the other five comparisons were less than 0.1, standing for different degrees of the publication bias. The trim-and-fill procedure was adopted for these 5 comparisons. After four additional studies were filled to “reoperation”, the result of the meta-analysis changed, and the OR changed from statistically to non-statistically significant, suggesting that DM as a risk factor for reoperation are not necessarily reliable and should be interpreted carefully. The other four comparisons (any complication, infection, readmission, and mortality) showed varying degree of changes in the pooled effect values after the adoption of trim-fill method, but without any change in the statistical significance (**Supplementary Figures S33–S38**).

## 4 Discussion

As the number of people with DM increases, a large number of diabetic patients are facing various health problems that require surgical treatment. DM is generally considered a major risk factor for postoperative complications (138). Although this is intuitive enough for clinicians, it is unclear which postoperative complications are exactly related to DM because there may be other comorbidities in patients with DM. This meta-analysis included 125 studies with a total sample size of 3,208,776.

We started out with a meta-analysis of any complication. The results showed that DM was a risk factor for any postoperative complication, which was consistent with previous studies (99). Our subgroup analyses showed that both IDDM and NIDDM were risk factors for any postoperative complication, and the risk of IDDM was higher than that of NIDDM. These findings suggested that IDDM, not just DM in general sense, should be an important risk factor in clinical evaluation of patients. This might explain why some diabetic patients, while their blood glucose was under control, still experienced various postoperative complications. The results of our meta-analysis are in accord with Nathan et al. study which found a 2.5-fold increase in the readmission rate of IDDM patients after posterior lumbar fusion. Subgroup analysis showed that readmission rate was nearly the same for patients with NIDDM as for those without DM, while it was twice as high in patients with IDDM as those without DM (65). Similar findings were reported in lumbar fusion surgery. Nicholas et al. suggested that compared with patients without DM, IDDM was more significantly associated with an increased risk of postoperative complications, extended length of hospital stay, postoperative adverse events, and readmission than NIDDM. Furthermore, the complications associated with IDDM were more severe than those associated with NIDDM (49). These findings indicated that whether a patient has IDDM is more important than whether a patient has DM (type 1 or type 2) when considering a patient as a surgical candidate.

It is well known that DM is a risk factor for perioperative complications (4, 46). Our analyses revealed that DM is an independent risk factor for wound infections, WHD, hematoma, and renal insufficiency. DM has been identified as a risk factor for postoperative infection and poor healing because of its vascular lesions and immune effects (139). DM present with neutrophilic dysfunction which increases the risk of infection by the pathogen and decreases healing capacity (52). DM is associated with tissue hypoxia and increased blood viscosity. This slows the inflammatory response, which in turn alters wound healing and increases the risk of infection, especially in the lower extremities (140–143). In addition, several factors prevent wound healing in patients with DM, including reduced angiogenesis, multiple growth factors, and impaired macrophage function (144). These may be responsible for postoperative complications of DM.

Moreover, we also found that DM can increase the incidence of postoperative renal insufficiency, which deserves our attention. After hip and knee arthroplasty, diabetic patients are 1.5 times more likely to develop acute renal failure than nondiabetic patients (92). After orthotopic liver transplantation, renal insufficiency is significantly higher in patients with preexisting DM than in patients without DM (59.7% vs. 20.2%, *P* < 0.001) (59). Considering the elevated incidence of postoperative renal insufficiency in diabetic patients, surgeons should pay more attention to postoperative fluid management, intraoperative hypotension anesthesia, and perioperative nephrotoxic medications.

A surprising finding in our study is that DM does not significantly increase the risk of VTE and MI. Diabetic patients are prone to hypercoagulable state due to abnormal regulation of coagulation-related plasma proteins caused by prolonged hyperglycemia. Type 2 DM is associated with an increased risk of thrombosis and cardiovascular disease. Therefore, it is also generally accepted that diabetic patients may be at an increased risk of postoperative thrombosis. For example, Rena et al. retrospectively reviewed 5,538 patients who underwent sleeve gastrectomy between January 1, 2008 and September 30, 2016, at 5 weight loss centers in the United States (80). They found that a personal history of malignancy and type 2 DM increased the risk of mesenteric vein thrombosis. However, many studies have shown different results. Ravinder et al. found that DM was not an independent risk factor for the venous thrombosis in various cosmetic procedures, although it was an independent risk factor for major complications, especially infections. The prevalence of DM did not differ significantly between the VTE and non-VTE groups (21) (0.9% vs 1.8%, *P* = 0.37). VTE and MI are deadly serious postoperative complications with not only high morbidity and mortality, but also prolonged hospital stay and high charges. Accurate identification of which patients are high risk for thromboembolism helps to take more targeted and appropriate preventive measures. A variety of surgeries were included in our study (cardiac surgery was not within the scope of our study). Eleven studies involved VTE. MI was reported in four studies. Our study suggests that DM is not a risk factor for postoperative VTE and MI, and therefore DM should not be considered a priority factor in determining thrombotic risk. Clinicians should pay more attention to age, smoking, and immobility and other factors, which may be associated with thrombosis according to the literature (116).

Our study also found that DM is a risk factor for postoperative reoperation and readmission, and that patients with DM have a higher risk of postoperative death. This is consistent with findings that DM is an independent risk factor for multiple postoperative complications. DM significantly prolongs the hospital stay after ankle fusion and total ankle replacement (104). Ganesh et al. used the NIS database to analyze the effect of DM on the prognosis of patients with ankle fractures and found that DM was associated with a significant increase in hospital stay (4.7 days vs 3.6 days, *P* < 0.001) (48). These are all consistent with our findings. Postoperative infection, hematoma and WHD are causes of reoperation and readmission. It is not surprising, therefore, that diabetic patients are more prone to reoperation and readmission. Although DM is found to be a risk factor for postoperative death, our subgroup analysis suggests that DM is a risk factor for death after orthopedic surgery but not cancer surgery, hemangioma resection and transplant. This is an important finding in our study. This may be related to the specifics of different types of surgery, such as patient population characteristics, length of surgery, and surgical procedures. Based on this finding, orthopedic surgery should be more strictly controlled by surgical standards and should be performed cautiously in patients with DM.

The strengths of this study lie in the large number of studies included the large sample size, and the exploration of the association of DM with multiple postoperative complications. The limitation of this study is that our study of common complications may have heterogeneity due to differences in the type of surgery. In addition, our combined effect size may be overestimated. The reason is that although a small number of included studies have analyzed a large number of complications, they only show significant differences (*P* < 0.05). Unfortunately, it is that we can only calculate the combined effect size based on studies that provide OR. Finally, what’s noteworthy is that the OR in some studies is not adjusted for confounders because the incidence of some complications may be affected by potential confounders, such as preoperative diseases other than DM, body weight, or age, etc. Therefore, we stratified the pooled values for any complication by crude OR and adjusted OR, respectively, resulting in consistent results. However, we did not stratify the other subdivided complications in the same way.

In summary, our meta-analysis suggested that DM may significantly affect multiple perioperative complications, hospitalization, and survival (cardiac surgery is not within the scope of our study). DM is a risk factor for postoperative infections, WHD, hematoma, renal insufficiency, reoperation, readmission and death after orthopedic surgery. But DM is not the risk of postoperative VTE, MI and not the risk for death after cancer surgery, hemangioma resection and transplant. These findings underscore the importance of preoperative risk factor assessment for the safe outcome of surgical patients.

## Data Availability Statement

The original contributions presented in the study are included in the article/**Supplementary Material**. Further inquiries can be directed to the corresponding authors.

## Author Contributions

XZ, AH, WM, and JL contributed to the conception or design the study; JC, YL, JSL, and HL contributed to acquisition, analysis of data for the study; XZ, AH, YM, and YS contributed to interpretation of data for the study; XZ and AH wrote the first draft of the manuscript. All authors revised it critically for important intellectual content and approved the final manuscript. All authors agreed to be accountable for all aspects of the work in ensuring that questions related to the accuracy or integrity of any part of the work are appropriately investigated and resolved.

## Funding

This work was supported by the National Key Research and Development Program of China (Grant No. 2018YFC2001900).

## Conflict of Interest

The authors declare that the research was conducted in the absence of any commercial or financial relationships that could be construed as a potential conflict of interest.

## Publisher’s Note

All claims expressed in this article are solely those of the authors and do not necessarily represent those of their affiliated organizations, or those of the publisher, the editors and the reviewers. Any product that may be evaluated in this article, or claim that may be made by its manufacturer, is not guaranteed or endorsed by the publisher.

## Abbreviations

CI, Confidence Intervals; DM, Diabetes mellitus; IDDM, Insulin-Dependent Diabetes Mellitus; LOS, length of stay; MI, Myocardial infarction; NIDDM, Non-Insulin-Dependent Diabetes Mellitus; NOS, Newcastle-Ottawa scale; OR, Odds Ratio; RR, Relative Risk; WHD, Wound Healing Disorders; VTE, Venous Thromboembolism.
