# Supplementary material for: Association of Diabetes Mellitus With Postoperative Complications and Mortality After Non-Cardiac Surgery: A Meta-Analysis and Systematic Review
Source: Front Endocrinol (Lausanne). 2022 May 26;13:841256. doi: 10.3389/fendo.2022.841256 (PMC9204286; doi:10.3389/fendo.2022.841256)
Supplement: Supplementary file 1 [file DataSheet_1.pdf]

Supplementary Material

Figure S1. Forest plot of odds ratio of any postoperative complication in patients with IDDM vs those without DM

Figure S2. Forest plot of odds ratio of any postoperative complication in patients with NIDDM vs those without DM

Figure S3. Forest plot of odds ratio of postoperative infections in patients with DM vs those without DM

Figure S4. Forest plot of odds ratio of postoperative venous thromboembolism in patients with DM vs those without DM

Figure S5. Forest plot of odds ratio of postoperative wound healing disorders in patients with DM vs those without DM

Figure S6. Forest plot of odds ratio of postoperative hematoma in patients with DM vs those without DM

Figure S7. Forest plot of odds ratio of postoperative renal insufficiency / failure in patients with DM vs those without DM

Figure S8. Forest plot of odds ratio of postoperative myocardial infarction in patients with DM vs those without DM

Figure S9. Forest plot of odds ratio of extended length of stay in patients with DM vs those without DM

Figure S10. Forest plot of odds ratio of reoperation in patients with DM vs those without DM

Figure S11. Forest plot of odds ratio of readmission in patients with DM vs those without DM

Figure S12. Forest plot of odds ratio of postoperative mortality after orthopedic surgery in patients with DM vs those without DM

Figure S13. Forest plot of odds ratio of postoperative mortality after cancer surgery in patients with DM vs those without DM

Figure S14. Forest plot of odds ratio of postoperative mortality after hemangioma resection in patients with DM vs those without DM

Figure S15. Forest plot of odds ratio of postoperative mortality after transplant in patients with DM vs those without DM

Figure S16. Forest plot of odds ratio of any postoperative complication in patients with DM vs those without DM in aesthetic surgery

Figure S17. Forest plot of odds ratio of postoperative infections in patients with DM vs those without DM in aesthetic surgery

Figure S18. Forest plot of odds ratio of postoperative venous thromboembolism in patients with DM vs those without DM in aesthetic surgery

Figure S19. Forest plot of odds ratio of postoperative hematoma in patients with DM vs those without DM in aesthetic surgery

Figure S20. Forest plot of odds ratio of any postoperative complication in patients with DM vs those without DM in general surgery

Figure S21. Forest plot of odds ratio of postoperative infections in patients with DM vs those without DM in general surgery

Figure S22. Forest plot of odds ratio of postoperative venous thromboembolism in patients with DM vs those without DM in general surgery

Figure S23. Forest plot of odds ratio of postoperative wound healing disorders in patients with DM vs those without DM in general surgery

Figure S24. Forest plot of odds ratio of postoperative renal insufficiency / failure in patients with DM vs those without DM in general surgery

Figure S25. Forest plot of odds ratio of postoperative mortality in patients with DM vs those without DM in general surgery

Figure S26. Forest plot of odds ratio of any postoperative complication in patients with DM vs those without DM in orthopedic surgery

Figure 27. Forest plot of odds ratio of postoperative infections in patients with DM vs those without DM in orthopedic surgery

Figure S28. Forest plot of odds ratio of postoperative venous thromboembolism in patients with DM vs those without DM in orthopedic surgery

Figure S29. Forest plot of odds ratio of postoperative wound healing disorders in patients with DM vs those without DM in orthopedic surgery

Figure S30. Forest plot of odds ratio of postoperative hematoma in patients with DM vs those without DM in orthopedic surgery

Figure S31. Forest plot of odds ratio of postoperative myocardial infarction in patients with DM vs those without DM in orthopedic surgery

Figure S32. Forest plot of odds ratio of postoperative mortality in patients with DM vs those without DM in general surgery in orthopedic surgery

Figure S33. Funnel plot of odds ratio of postoperative venous thromboembolism in patients with DM vs those without DM

Figure S34. Funnel plot of odds ratio of reoperation in patients with DM vs those without DM

Figure S35. Funnel plot of odds ratio of any postoperative complication in patients with DM vs those without DM

Figure S36. Funnel plot of odds ratio of postoperative infection in patients with DM vs those without DM

Figure S37. Funnel plot of odds ratio of readmission in patients with DM vs those without DM

Figure S38. Funnel plot of odds ratio of postoperative mortality in patients with DM vs those without DM

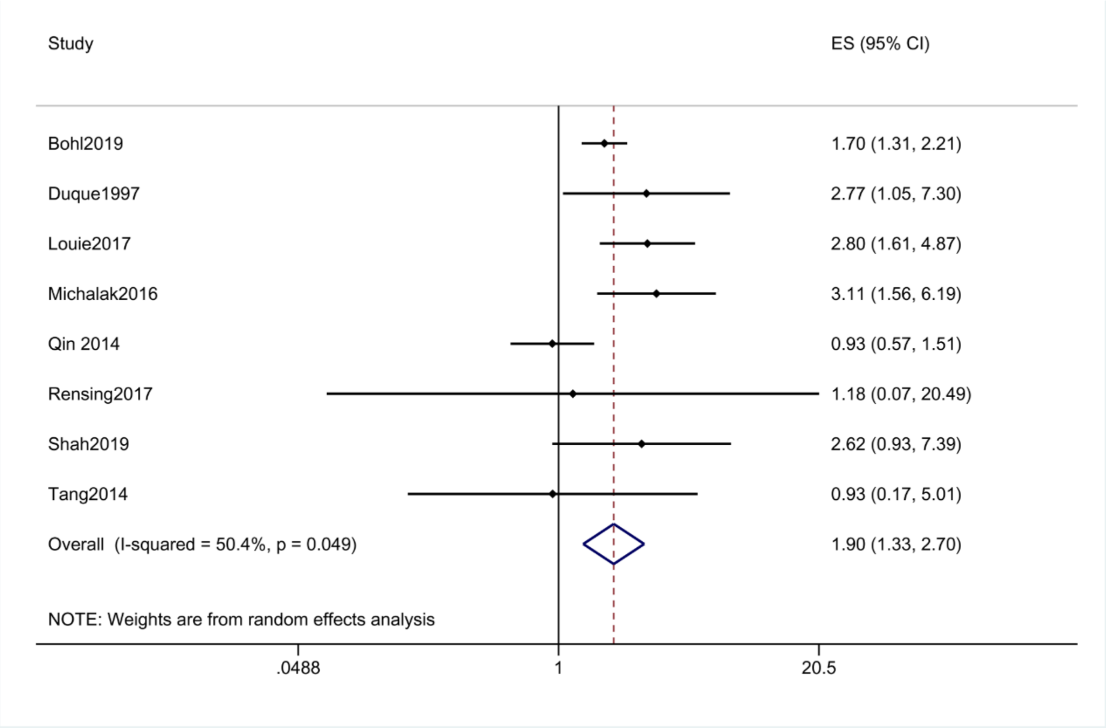

Figure S1. Forest plot of odds ratio of any postoperative complication in patients with IDDM vs those without DM

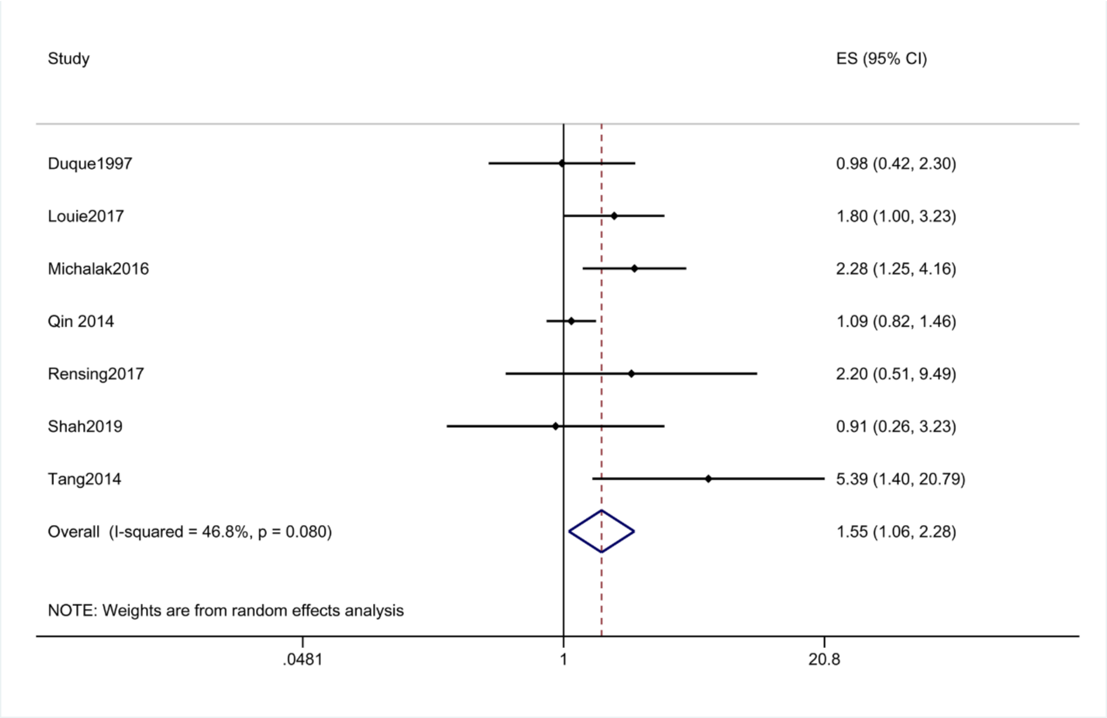

Figure S2. Forest plot of odds ratio of any postoperative complication in patients with NIDDM vs those without DM

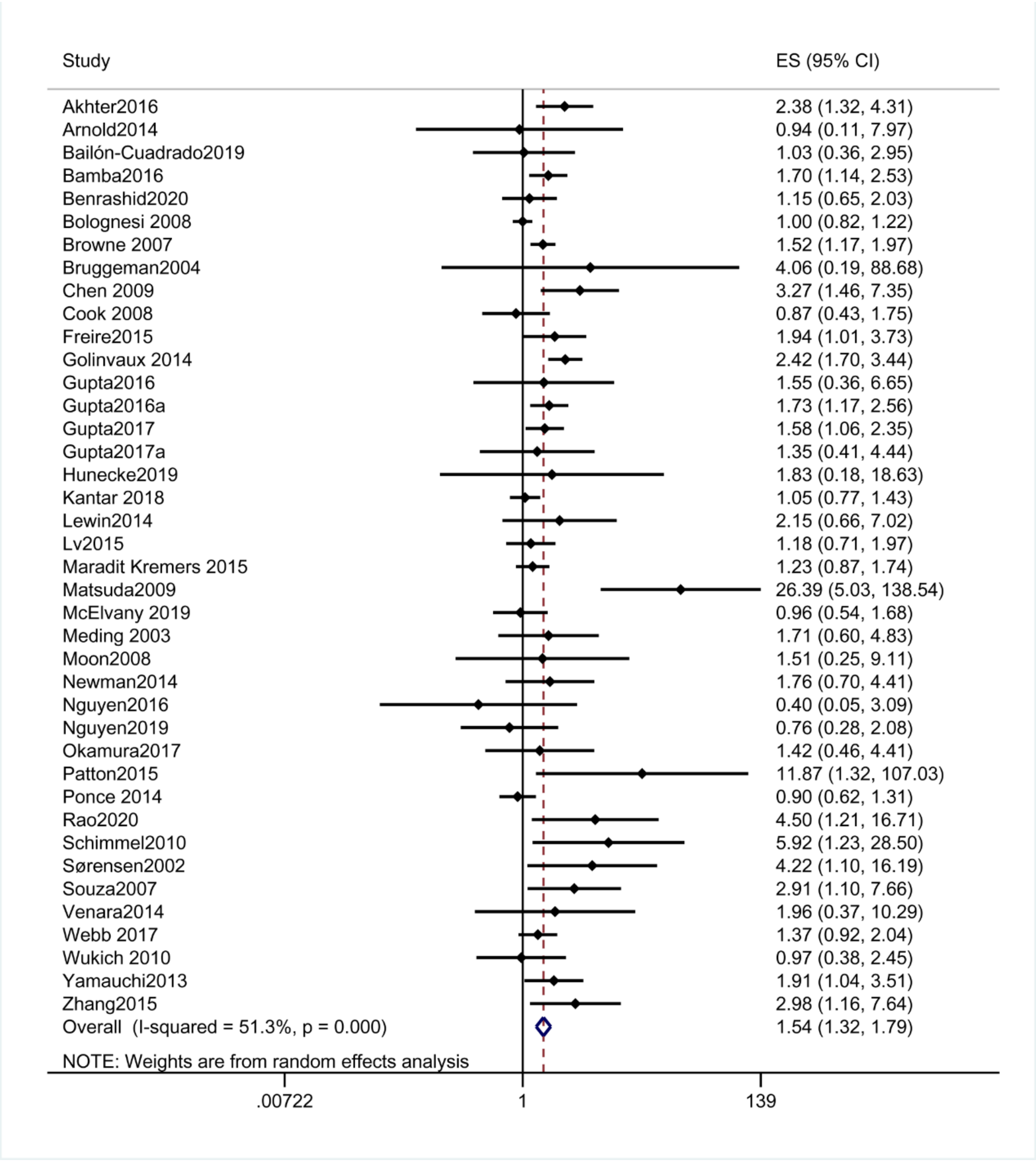

Figure S3. Forest plot of odds ratio of postoperative infections in patients with DM vs those without DM

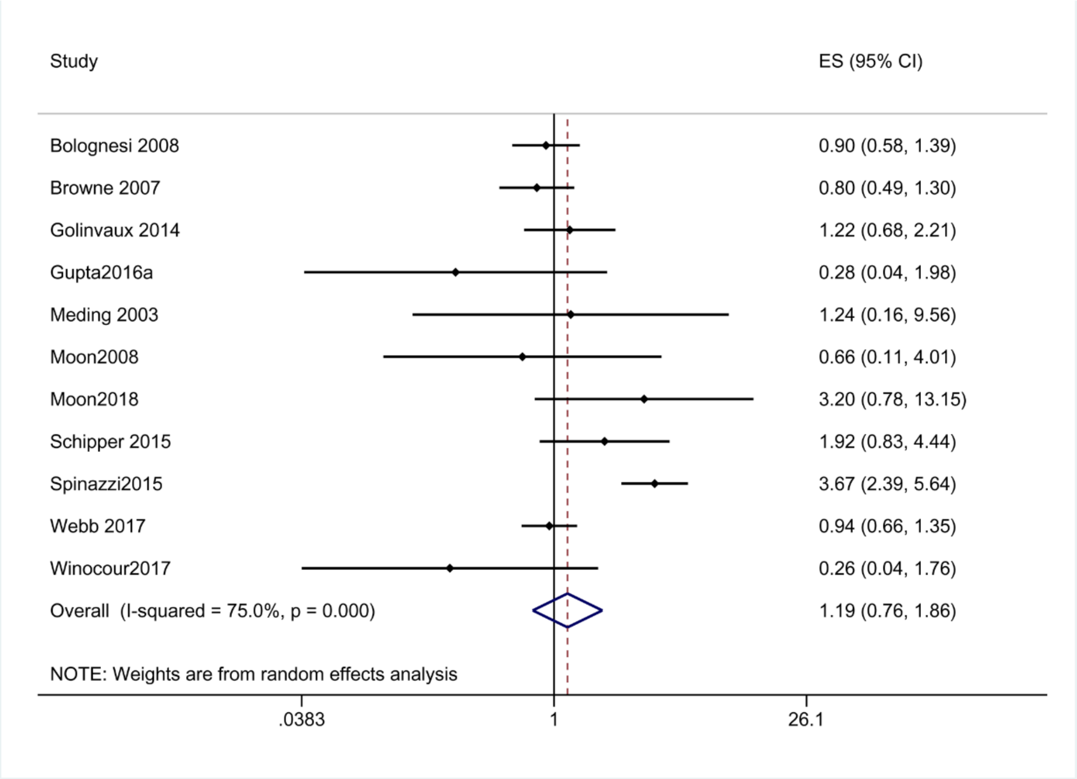

Figure S4. Forest plot of odds ratio of postoperative venous thromboembolism in patients with DM vs those without DM

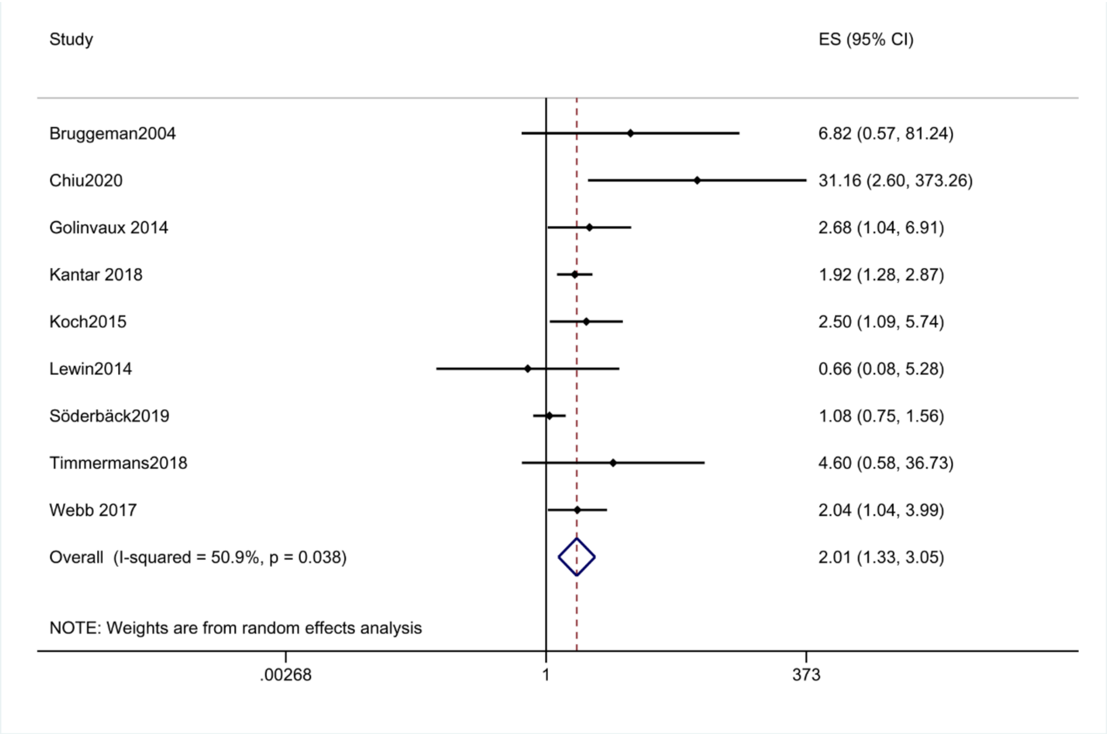

Figure S5. Forest plot of odds ratio of postoperative wound healing disorders in patients with DM vs those without DM

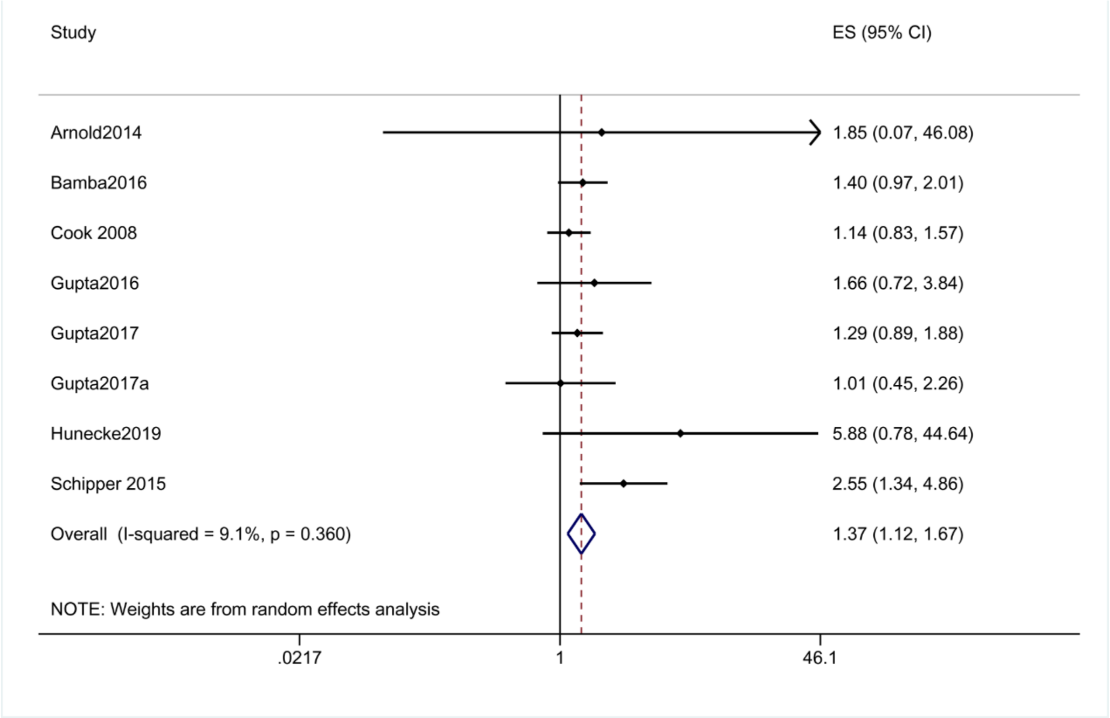

Figure S6. Forest plot of odds ratio of postoperative hematoma in patients with DM vs those without DM

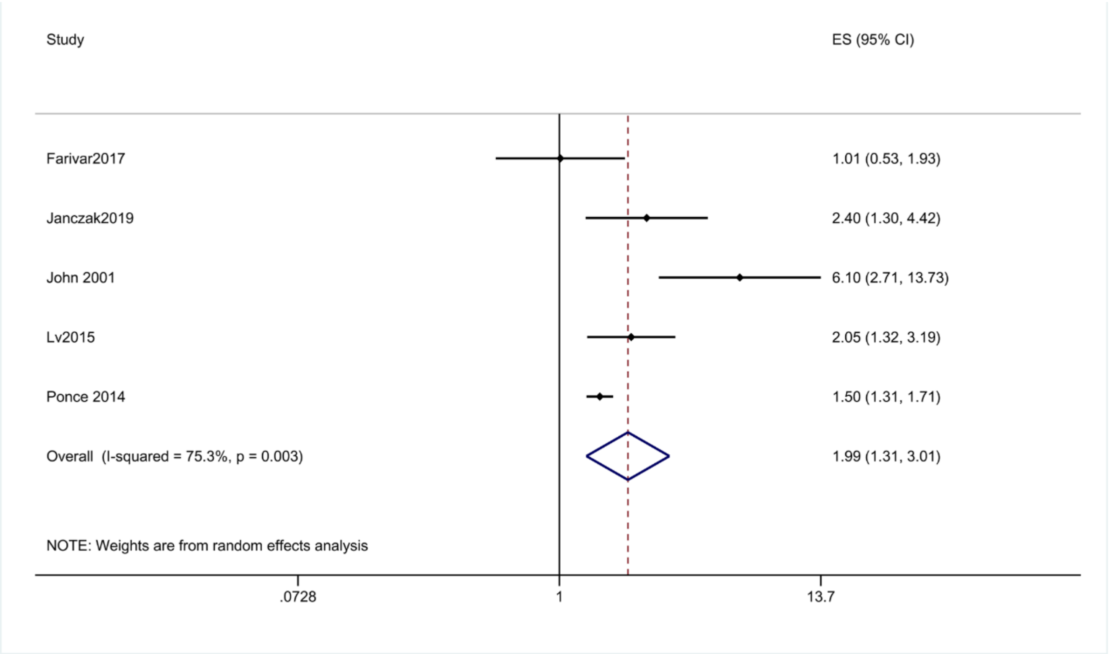

Figure S7. Forest plot of odds ratio of postoperative renal insufficiency / failure in patients with DM vs those without DM

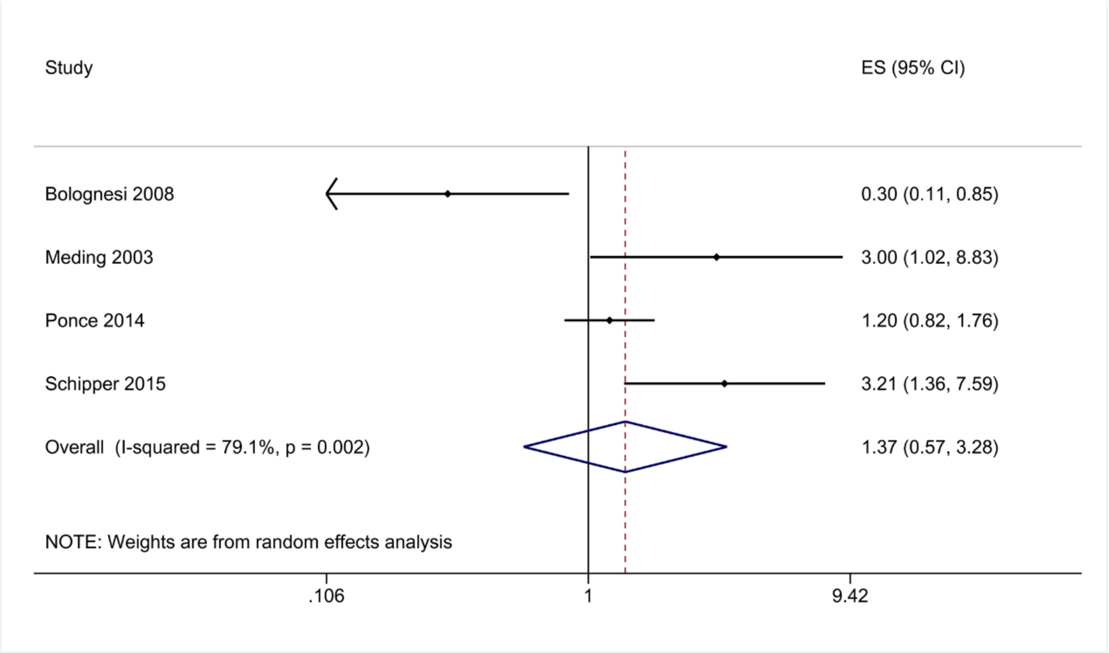

Figure S8. Forest plot of odds ratio of postoperative myocardial infarction in patients with DM vs those without DM

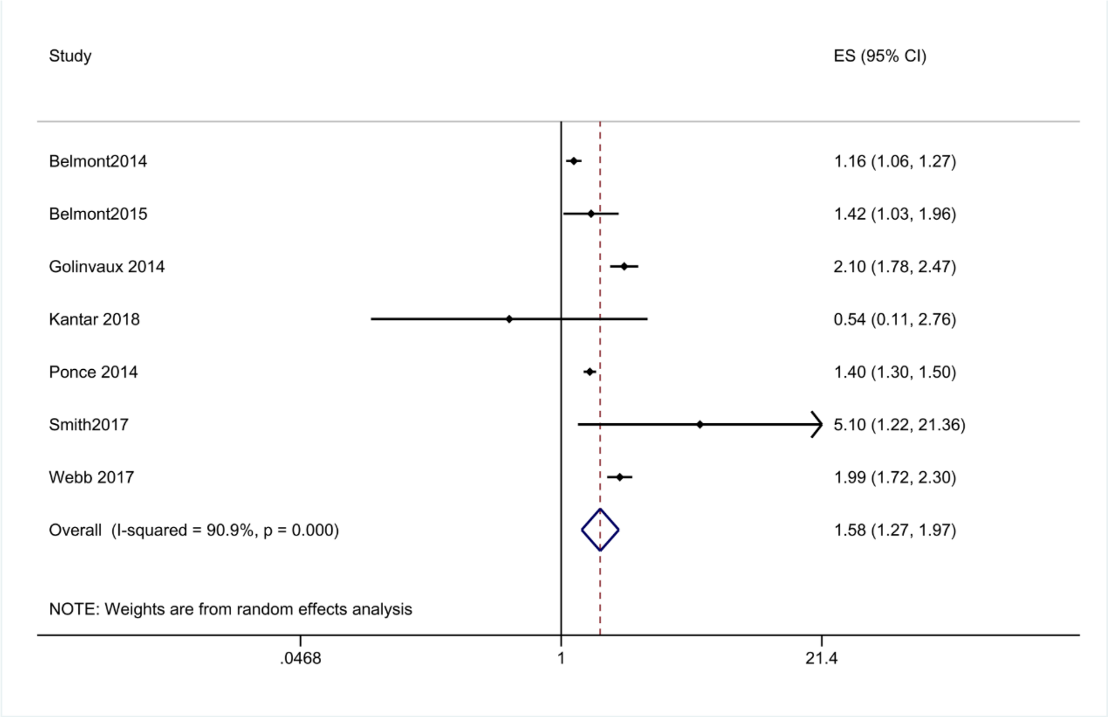

Figure S9. Forest plot of odds ratio of extended length of stay in patients with DM vs those without DM

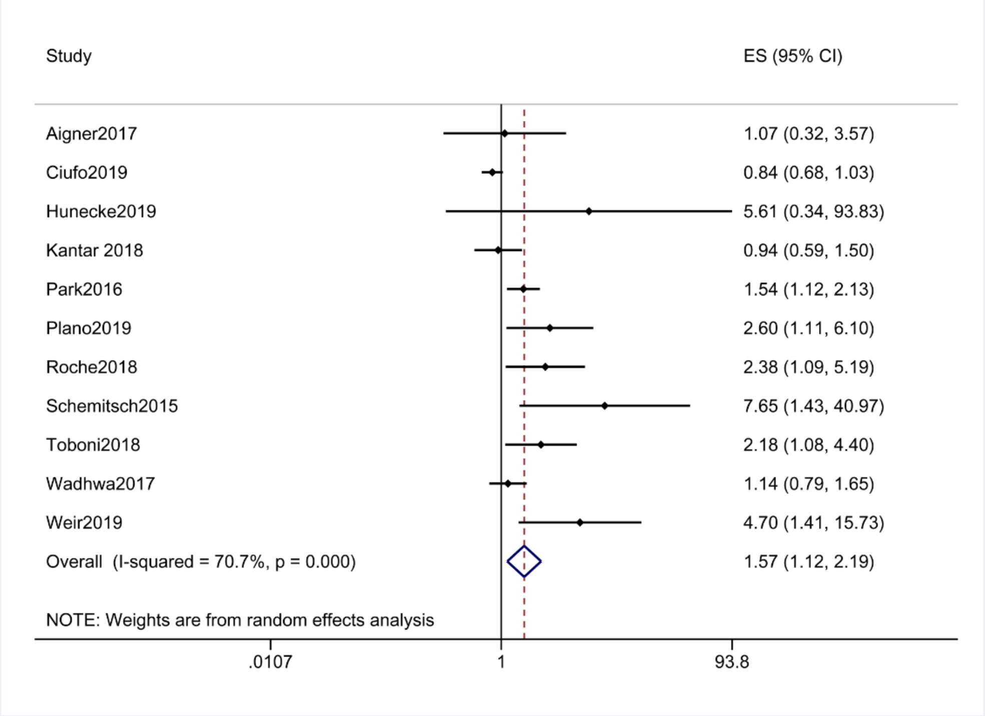

Figure S10. Forest plot of odds ratio of reoperation in patients with DM vs those without DM

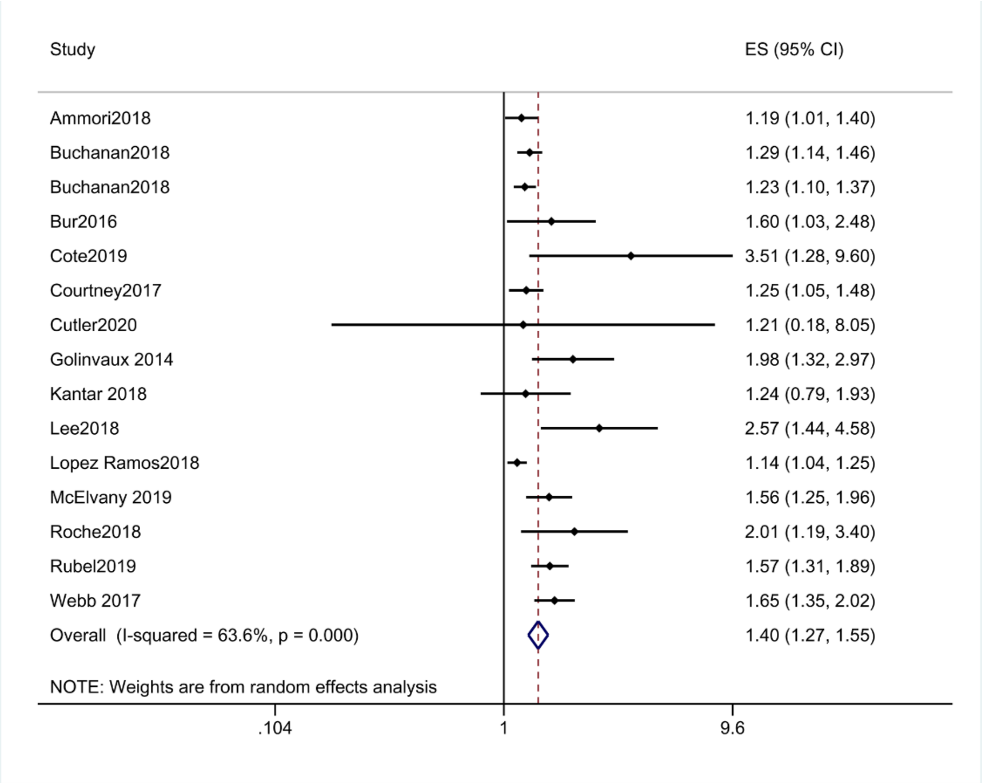

Figure S11. Forest plot of odds ratio of readmission in patients with DM vs those without DM

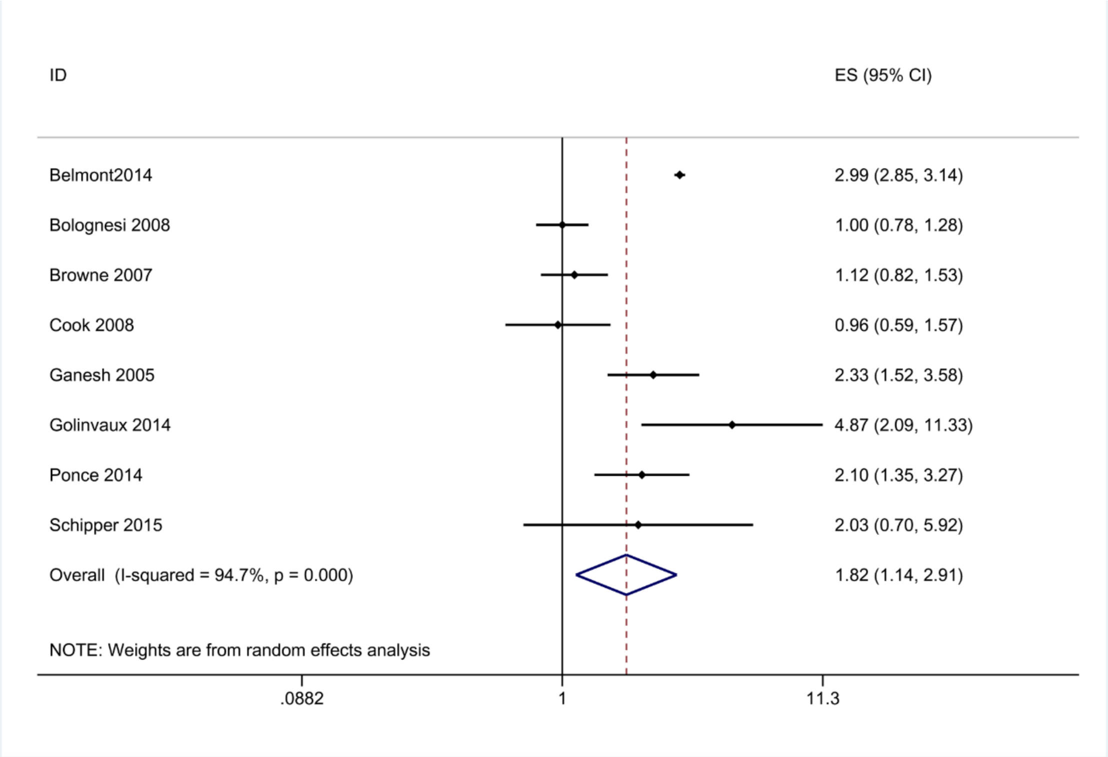

Figure S12. Forest plot of odds ratio of postoperative mortality after orthopedic surgery in patients with DM vs those without DM

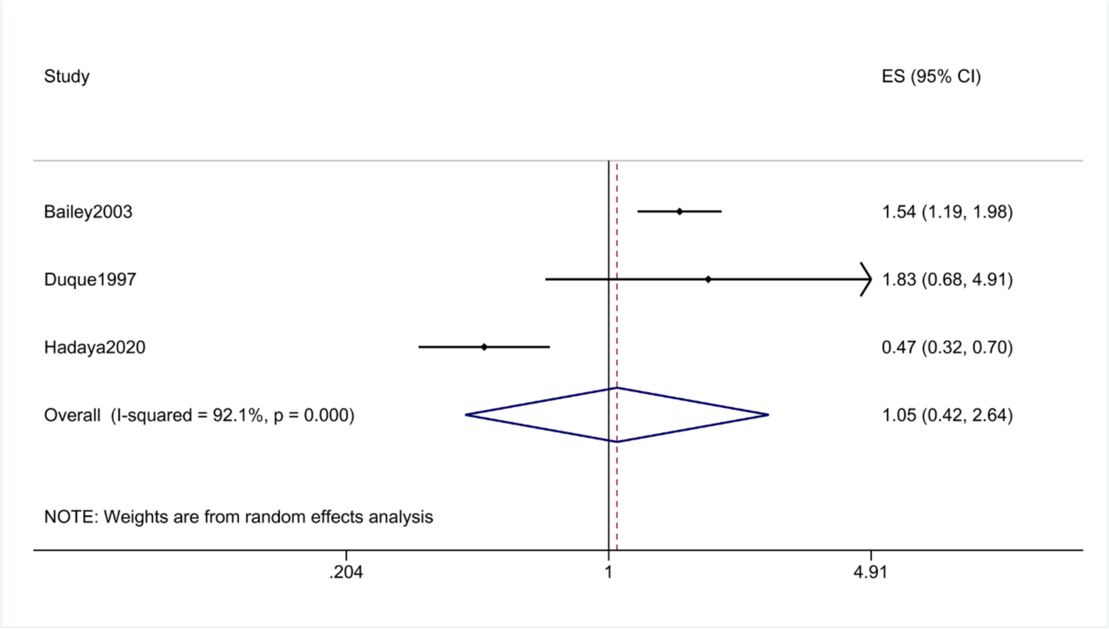

Figure S13. Forest plot of odds ratio of postoperative mortality after cancer surgery in patients with DM vs those without DM

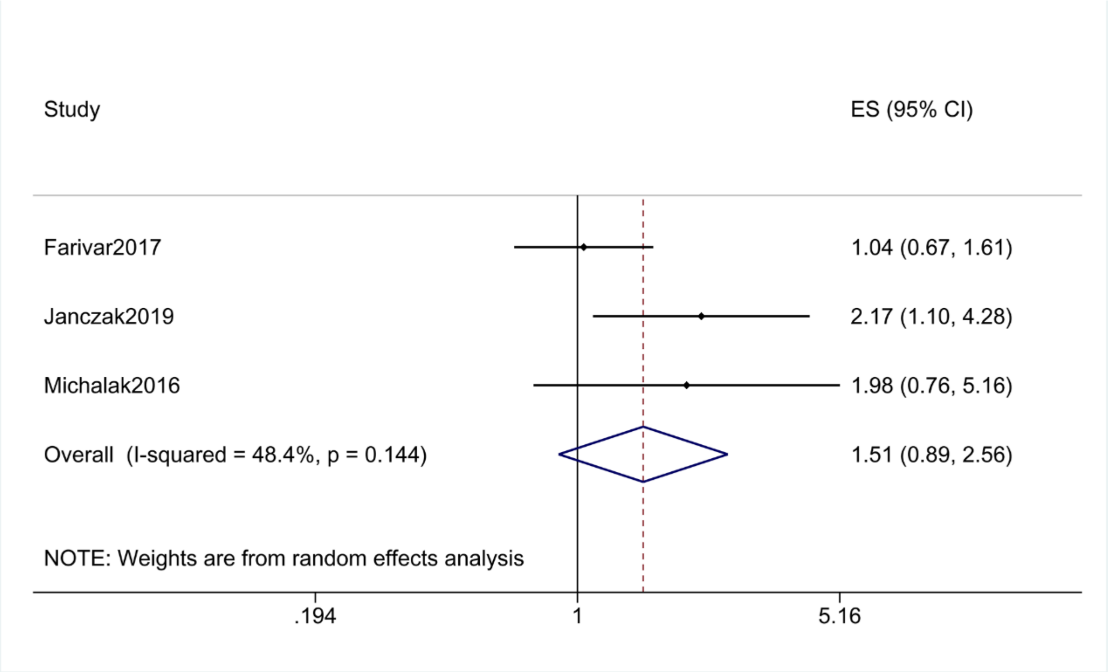

Figure S14. Forest plot of odds ratio of postoperative mortality after hemangioma resection in patients with DM vs those without DM

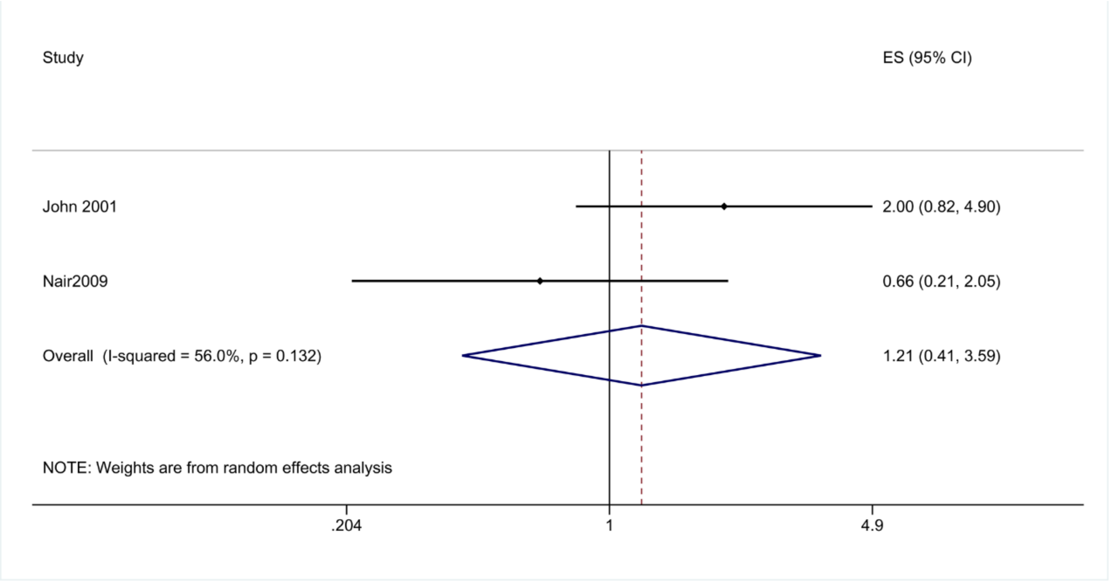

Figure S15. Forest plot of odds ratio of postoperative mortality after transplant in patients with DM vs those without DM

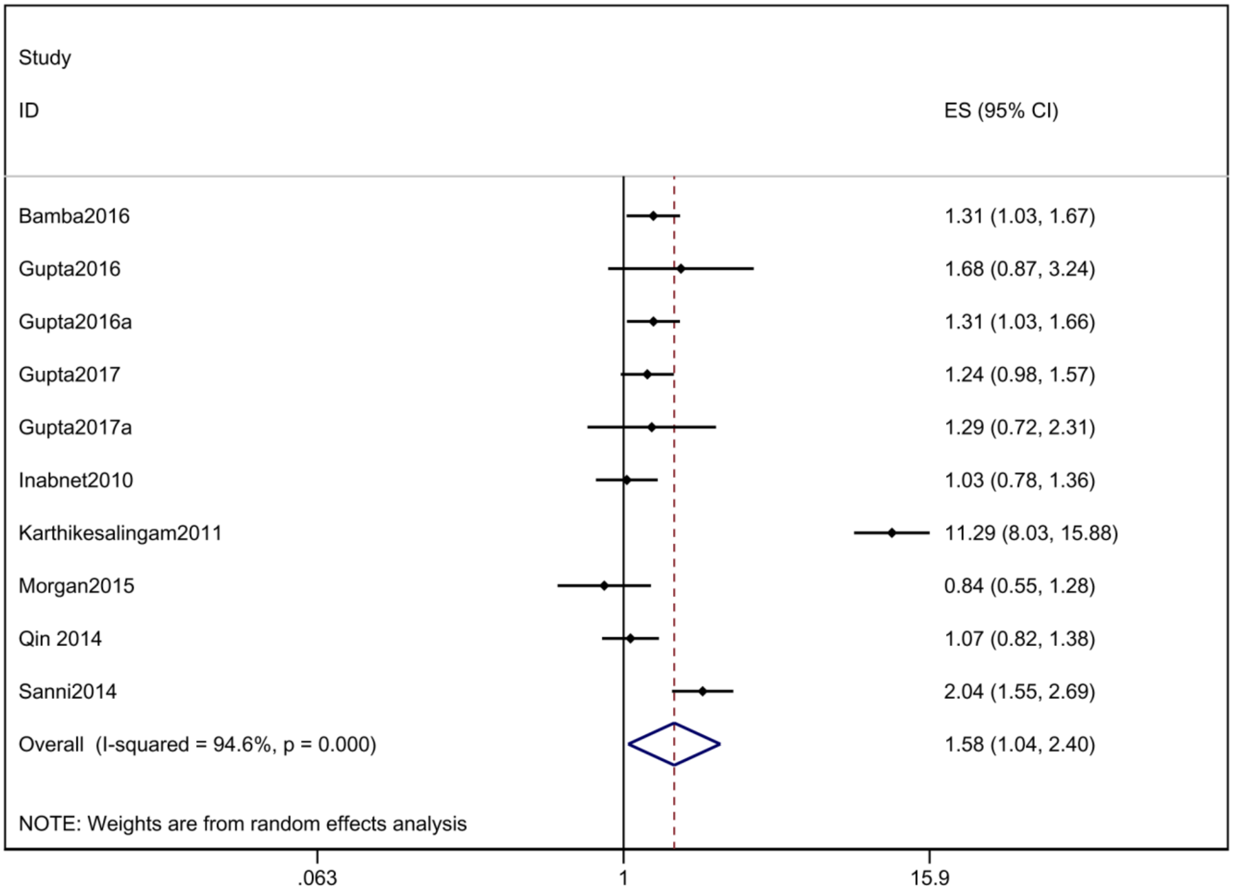

Figure S16. Forest plot of odds ratio of any postoperative complication in patients with DM vs those without DM in aesthetic surgery

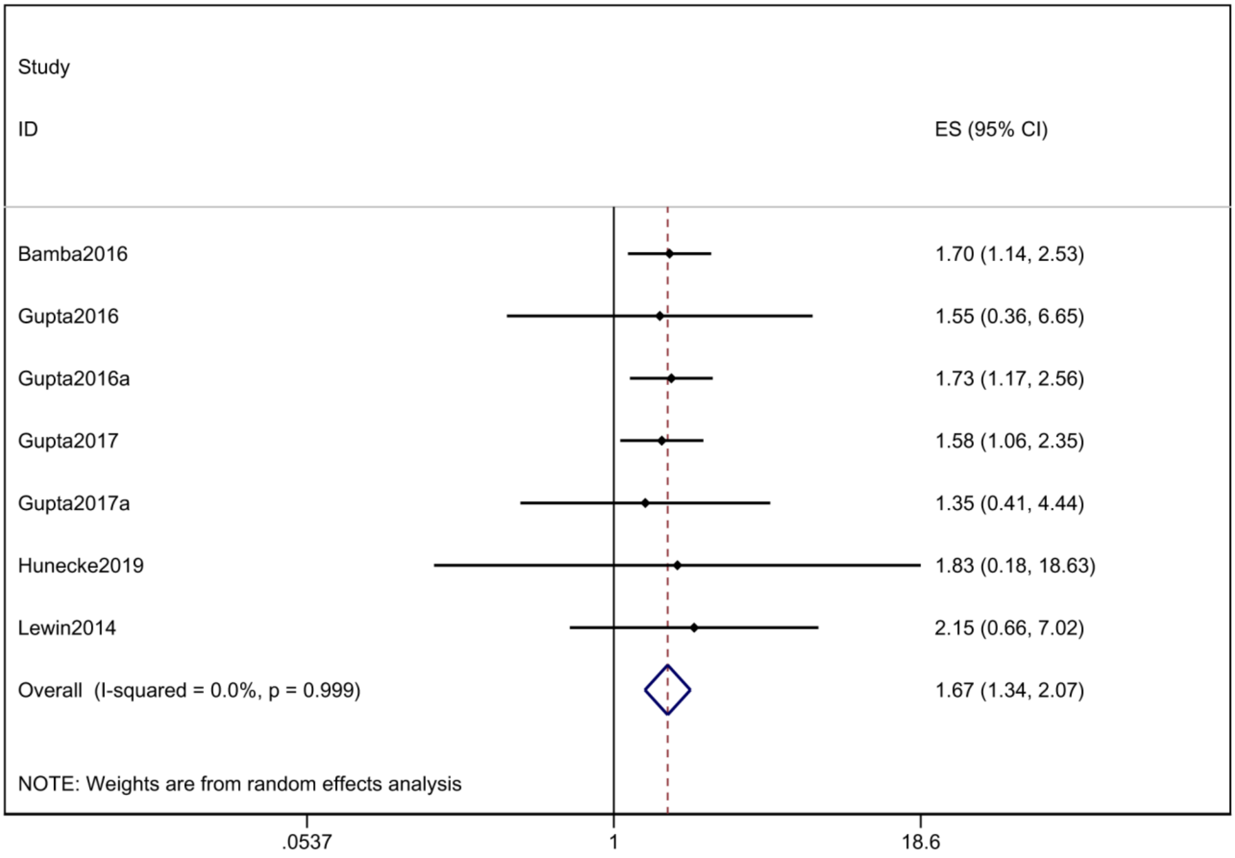

Figure S17. Forest plot of odds ratio of postoperative infections in patients with DM vs those without DM in aesthetic surgery

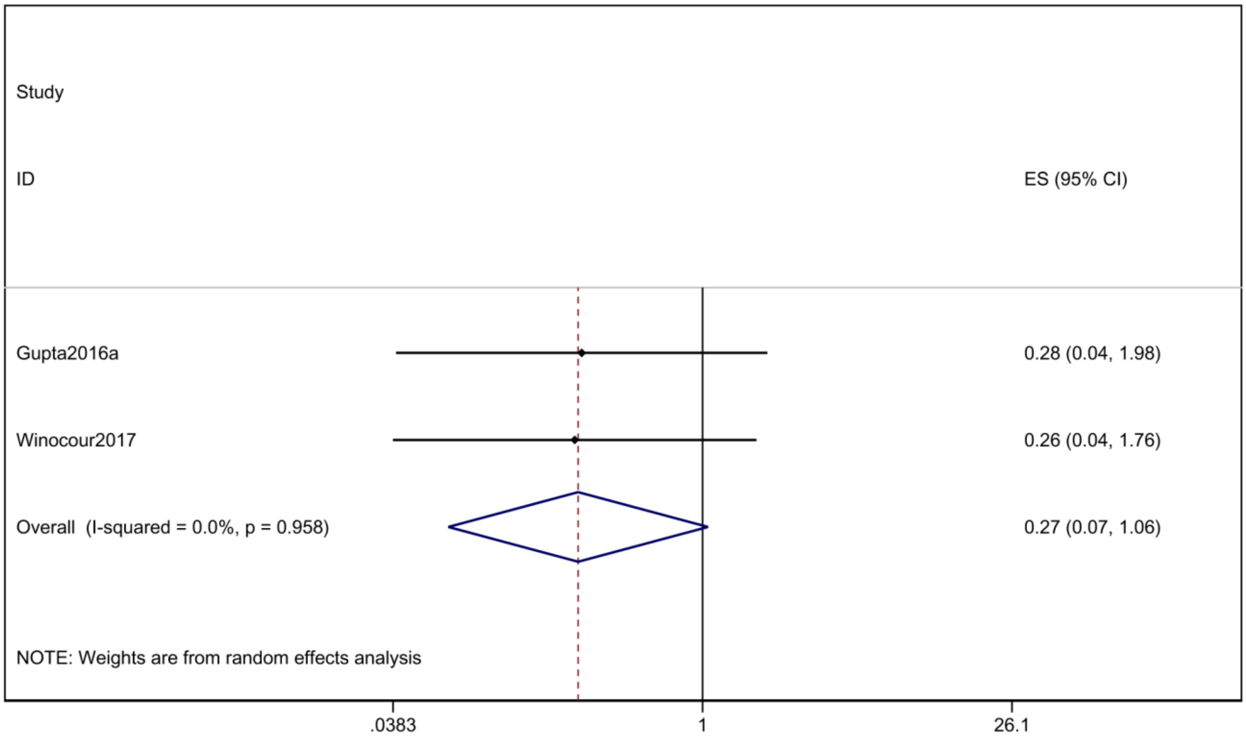

Figure S18. Forest plot of odds ratio of postoperative venous thromboembolism in patients with DM vs those without DM in aesthetic surgery

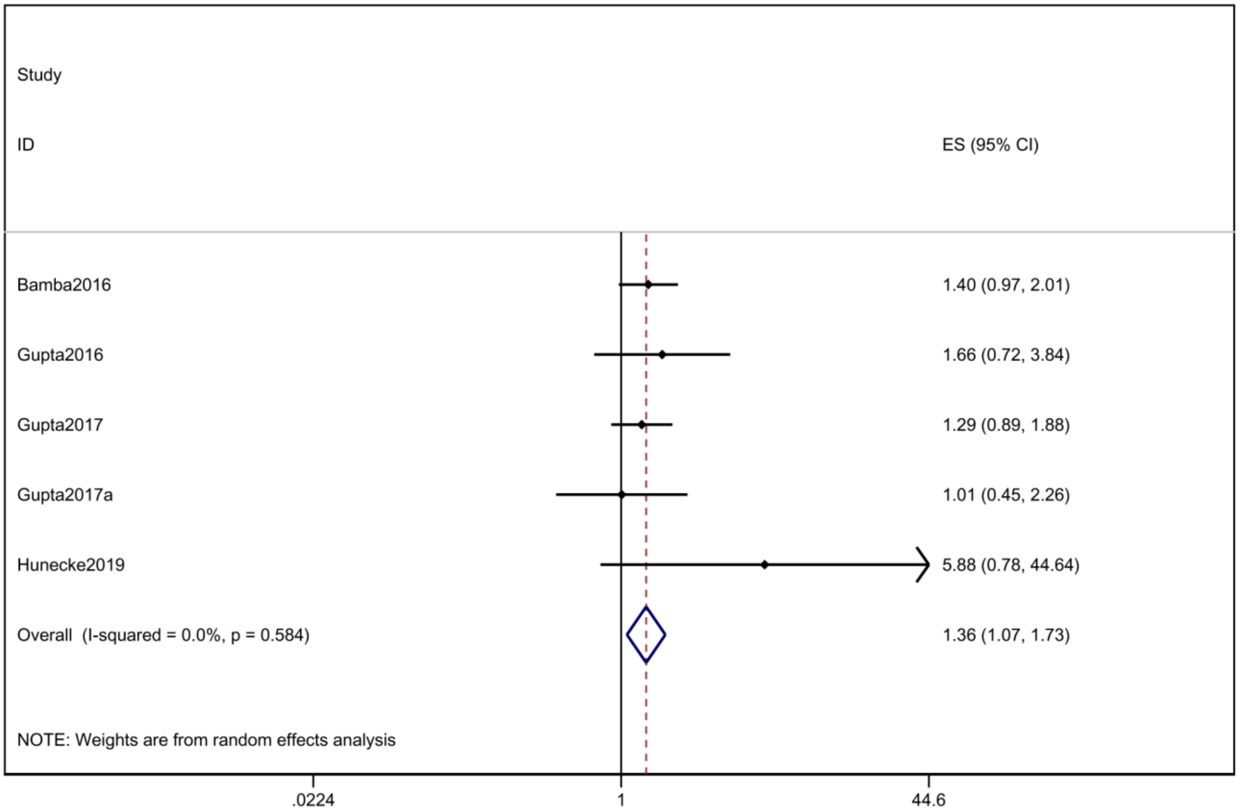

Figure S19. Forest plot of odds ratio of postoperative hematoma in patients with DM vs those without DM in aesthetic surgery

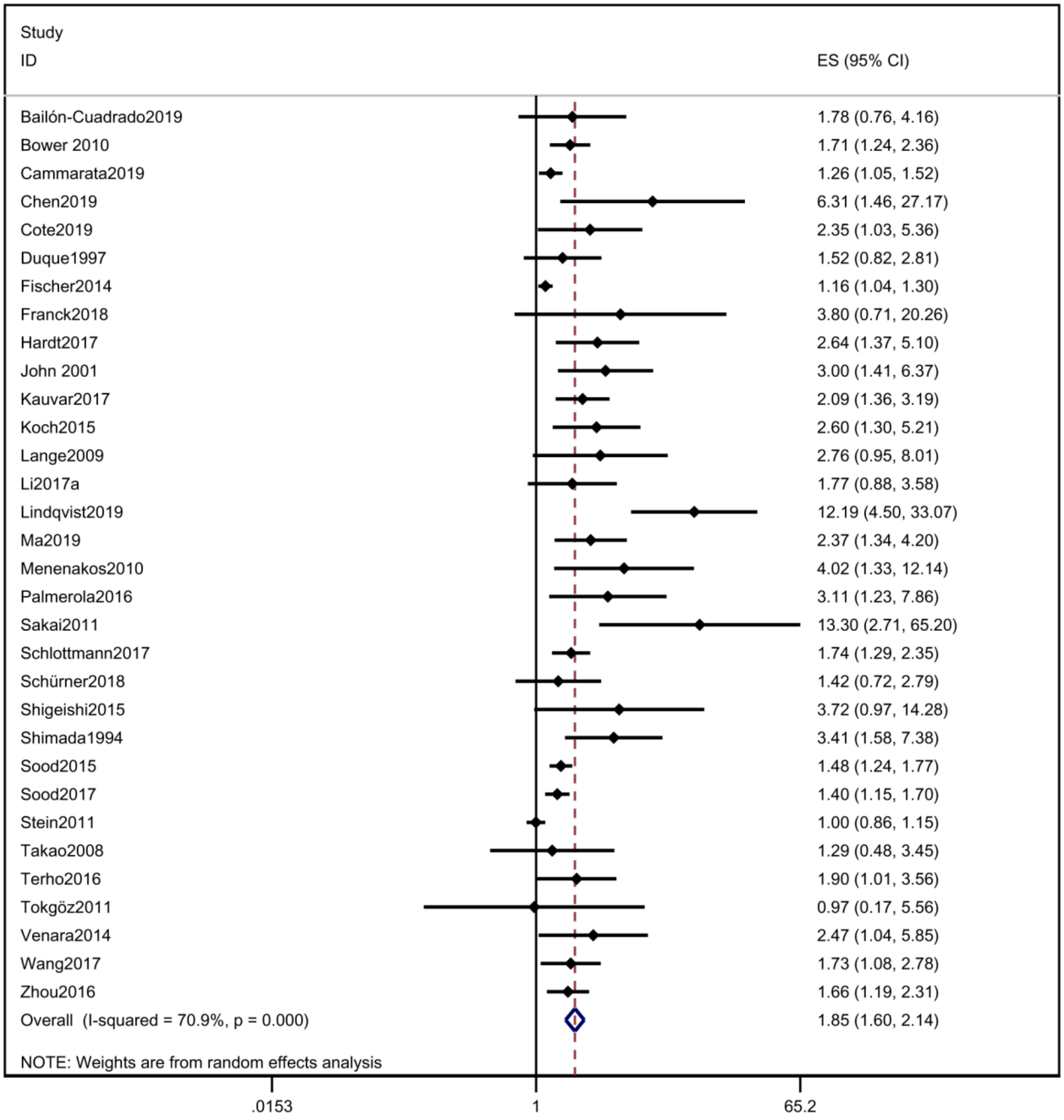

Figure S20. Forest plot of odds ratio of any postoperative complication in patients with DM vs those without DM in general surgery

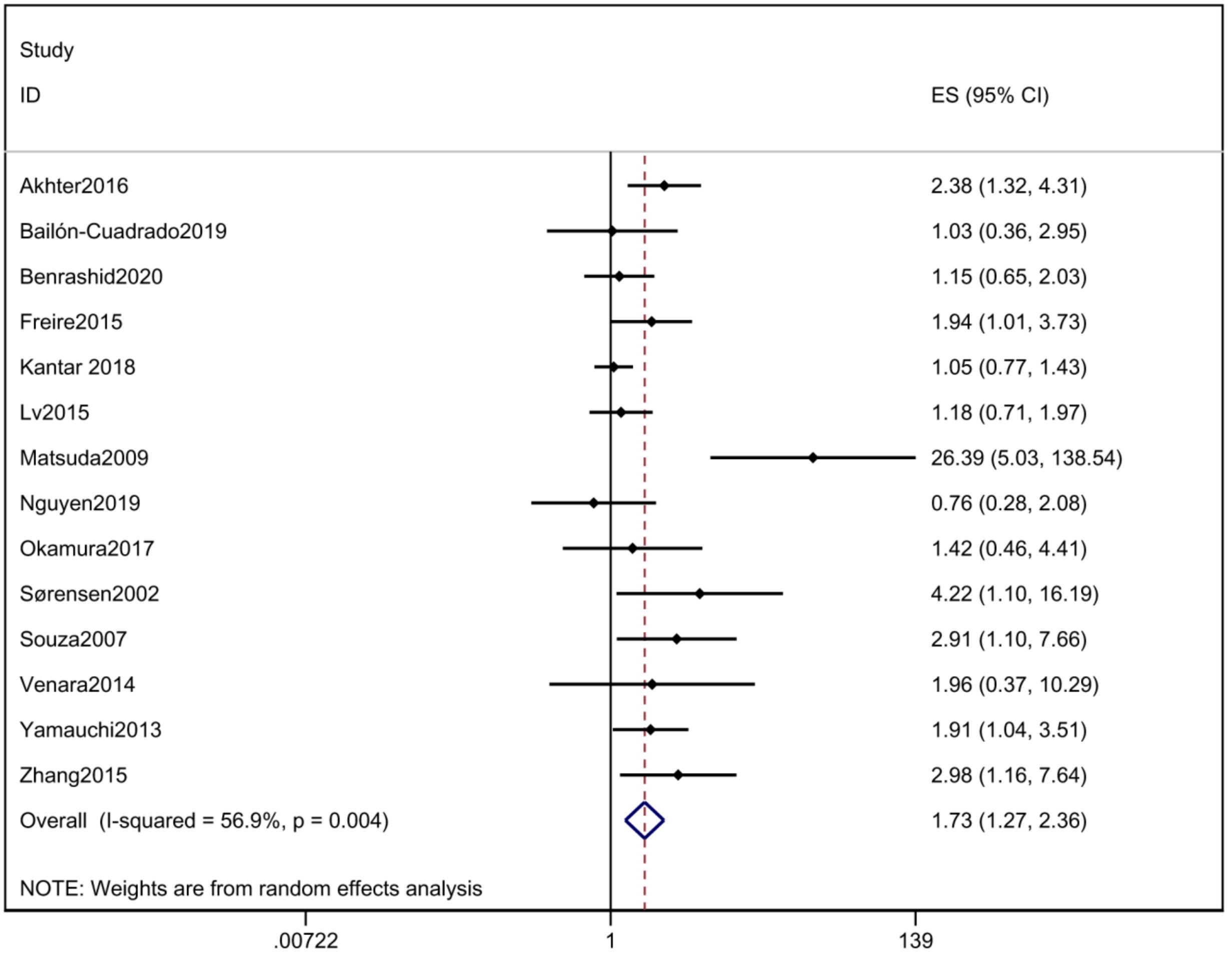

Figure S21. Forest plot of odds ratio of postoperative infections in patients with DM vs those without DM in general surgery

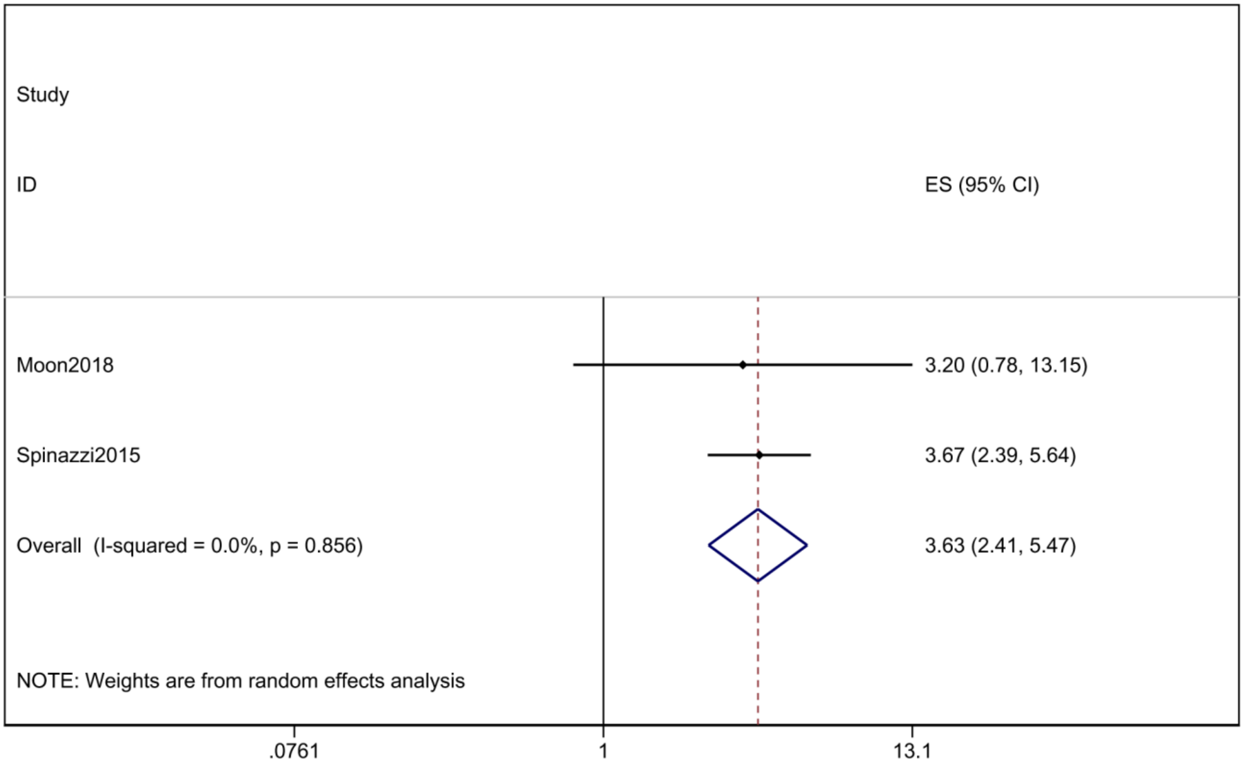

Figure S22. Forest plot of odds ratio of postoperative venous thromboembolism in patients with DM vs those without DM in general surgery

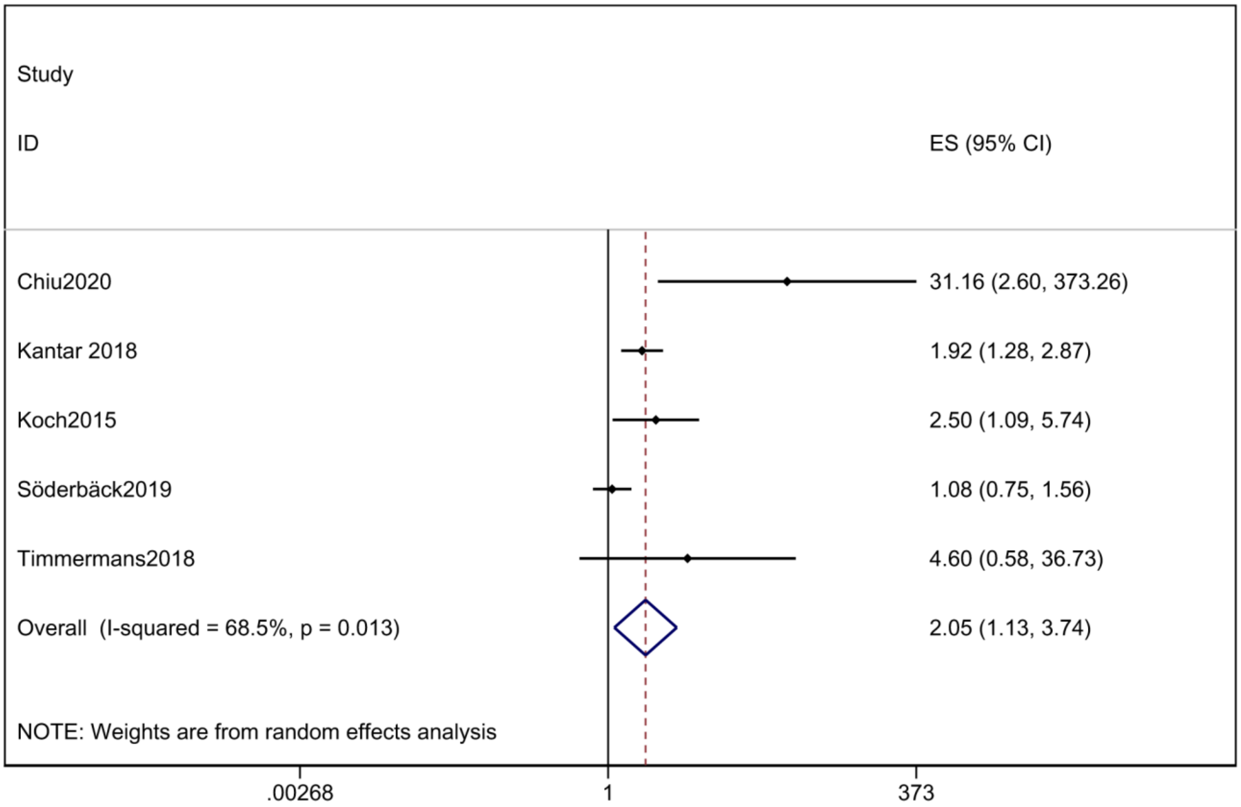

Figure S23. Forest plot of odds ratio of postoperative wound healing disorders in patients with DM vs those without DM in general surgery

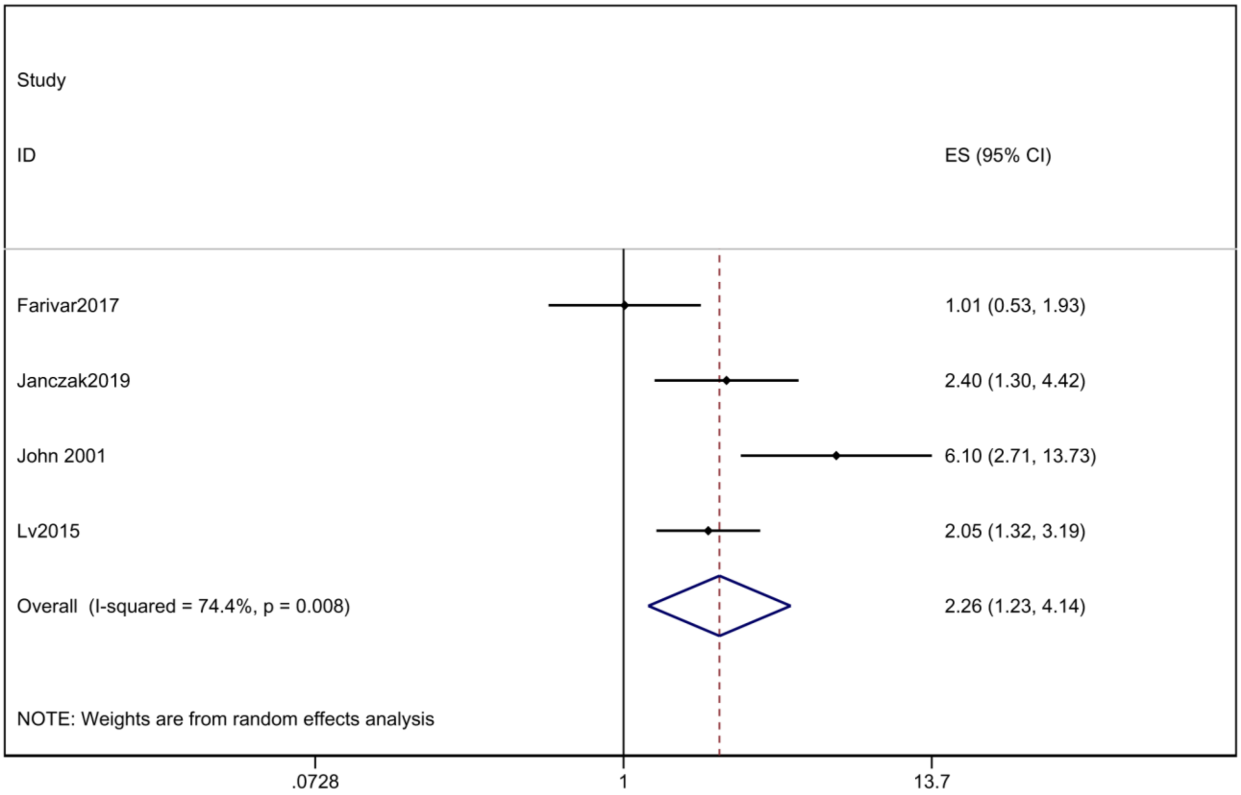

Figure S24. Forest plot of odds ratio of postoperative renal insufficiency / failure in patients with DM vs those without DM in general surgery

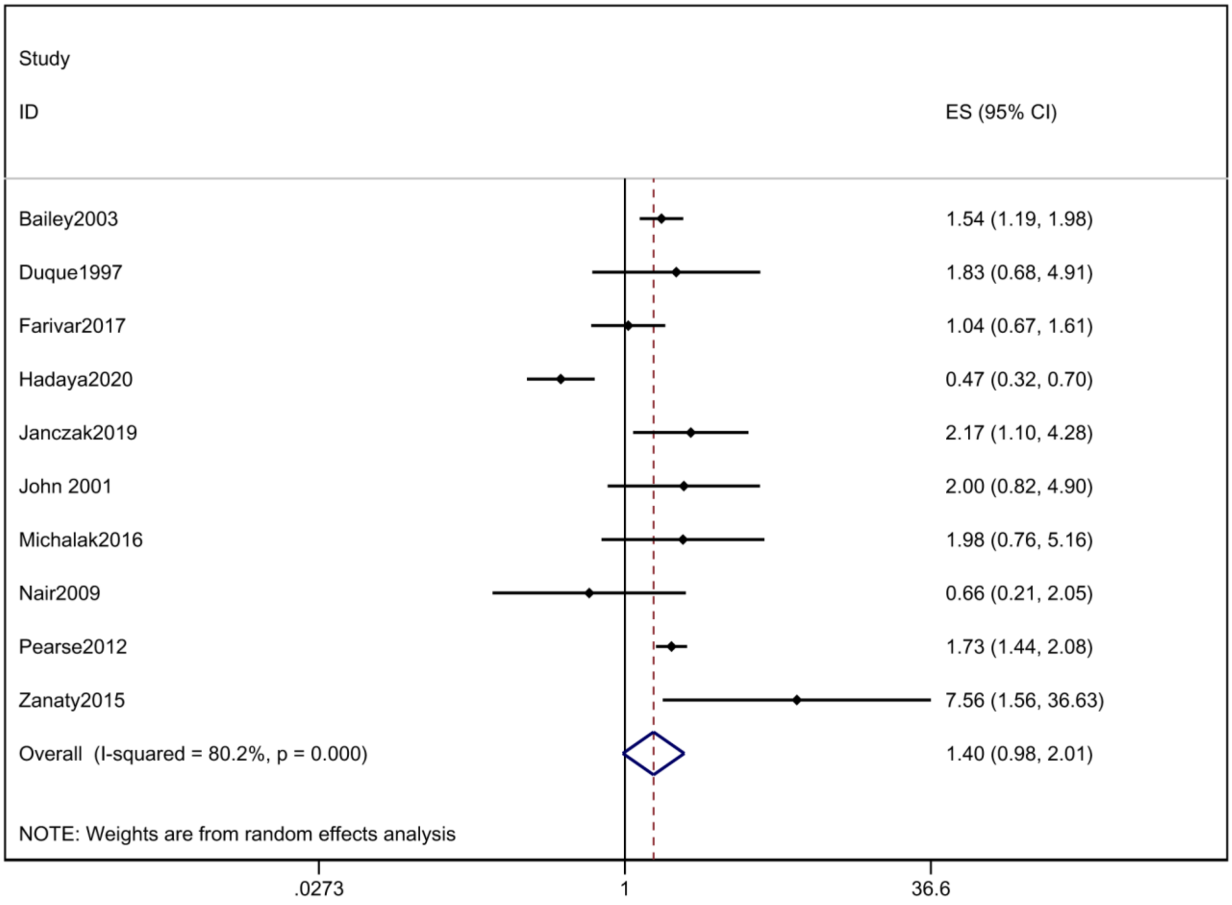

Figure S25. Forest plot of odds ratio of postoperative mortality in patients with DM vs those without DM in general surgery

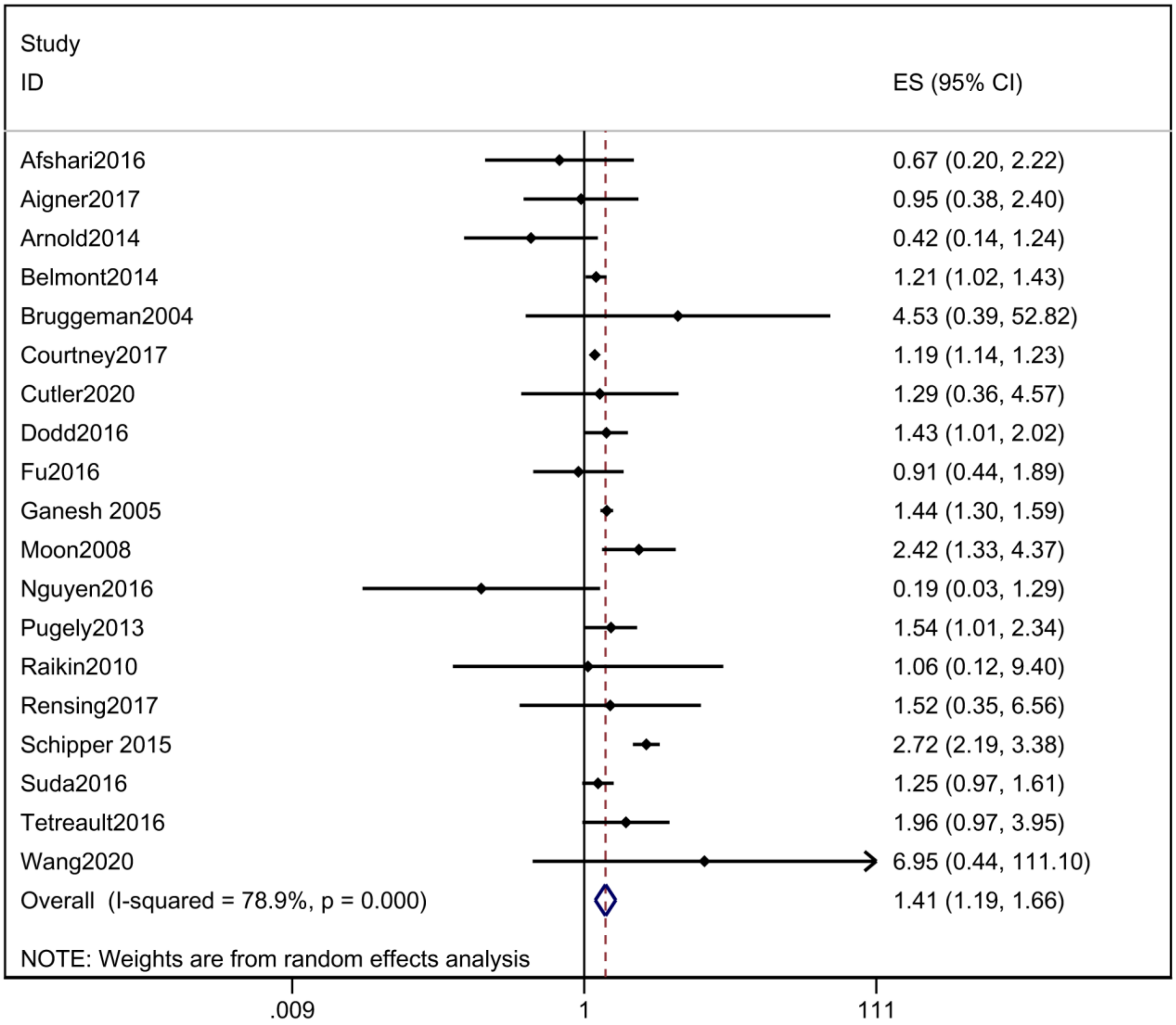

Figure S26. Forest plot of odds ratio of any postoperative complication in patients with DM vs those without DM in orthopedic surgery

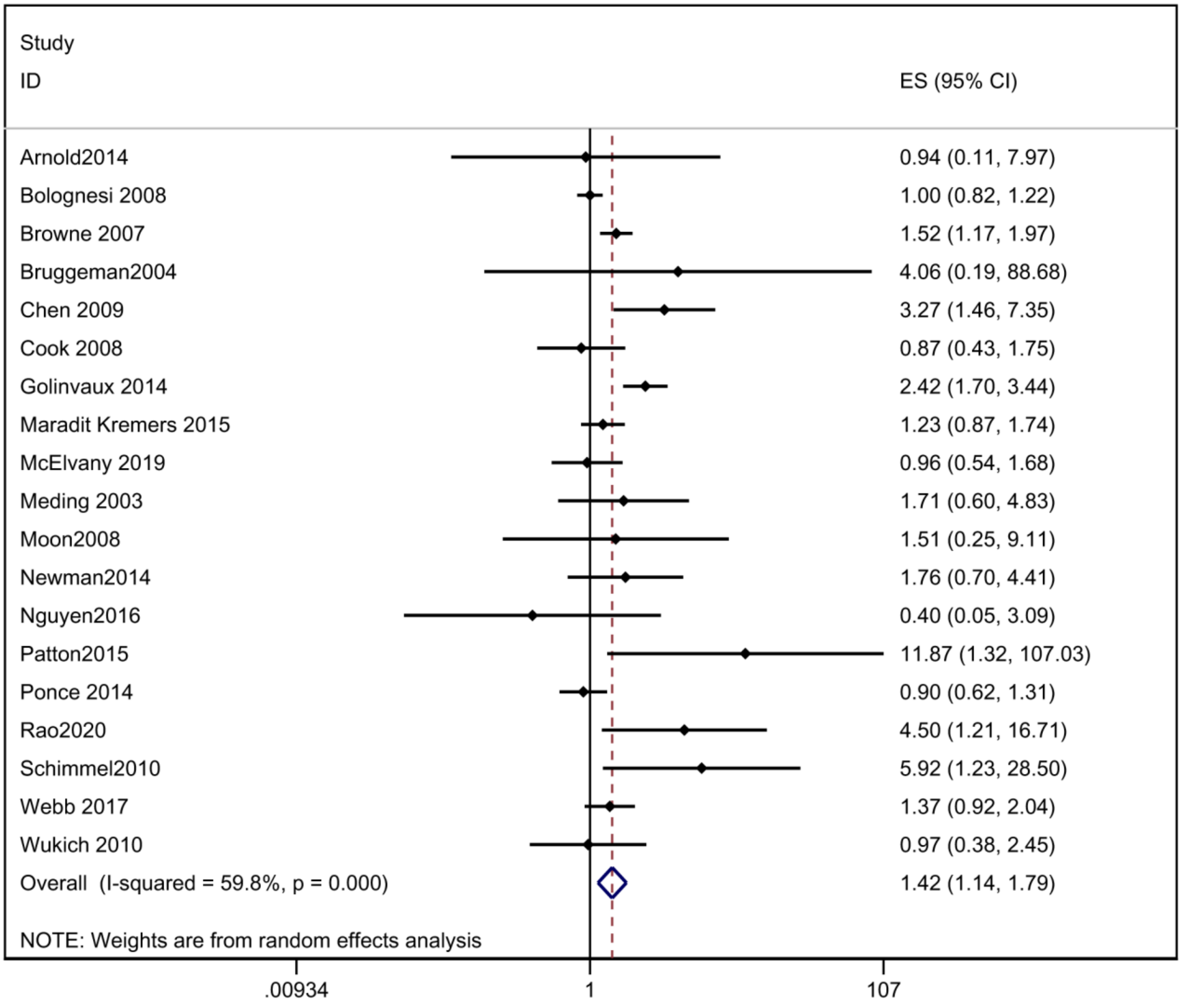

Figure 27. Forest plot of odds ratio of postoperative infections in patients with DM vs those without DM in orthopedic surgery

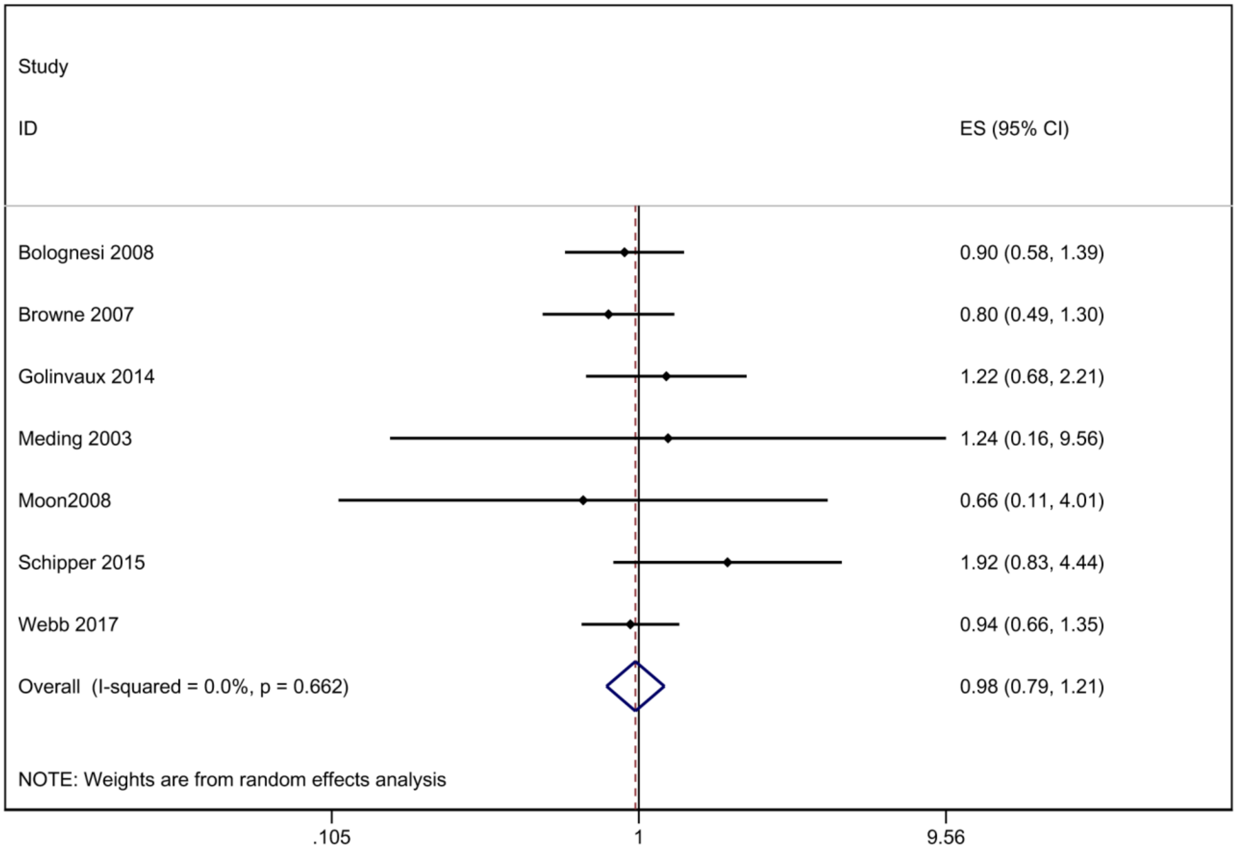

Figure S28. Forest plot of odds ratio of postoperative venous thromboembolism in patients with DM vs those without DM in orthopedic surgery

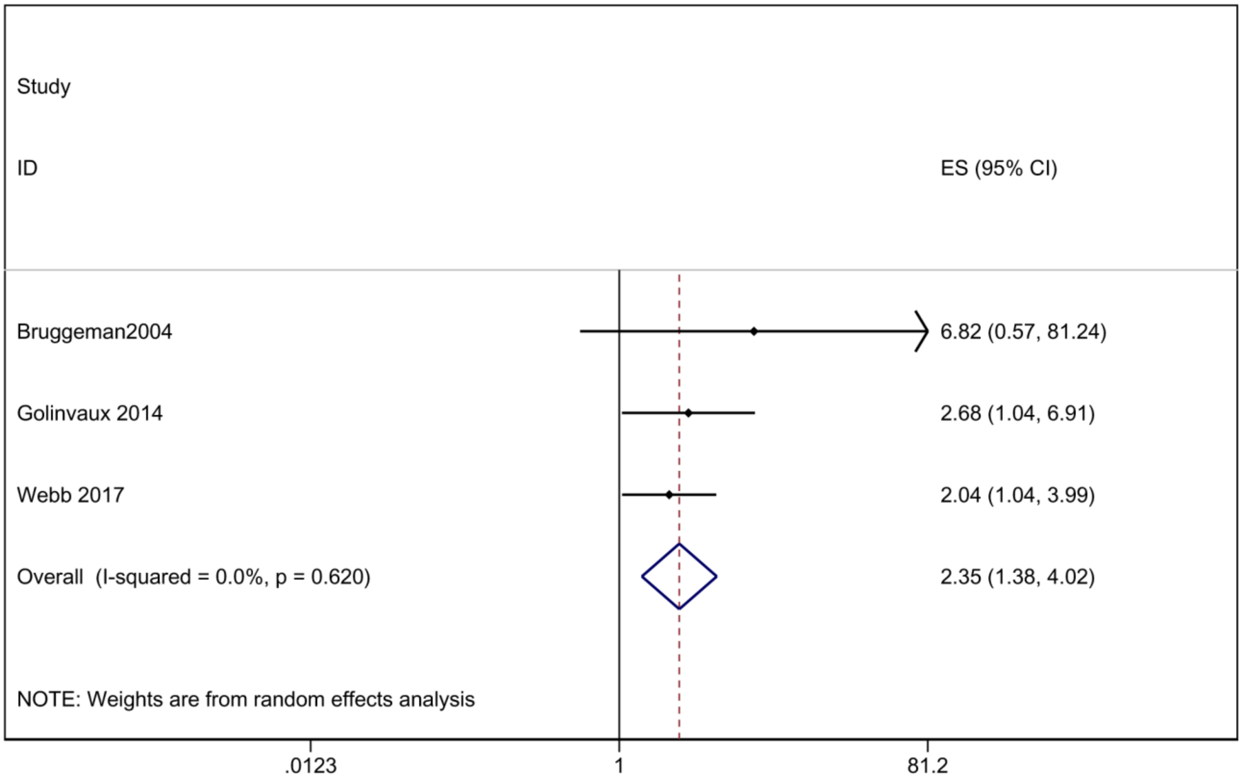

Figure S29. Forest plot of odds ratio of postoperative wound healing disorders in patients with DM vs those without DM in orthopedic surgery

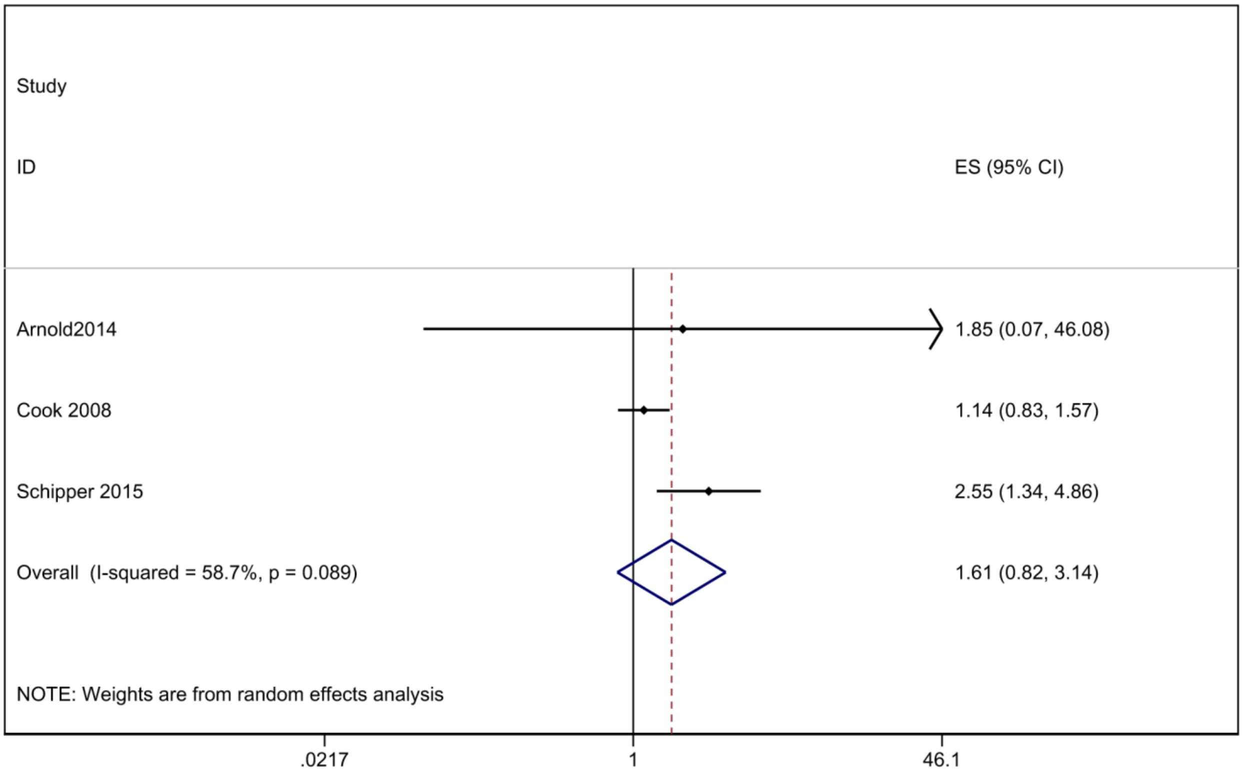

Figure S30. Forest plot of odds ratio of postoperative hematoma in patients with DM vs those without DM in orthopedic surgery

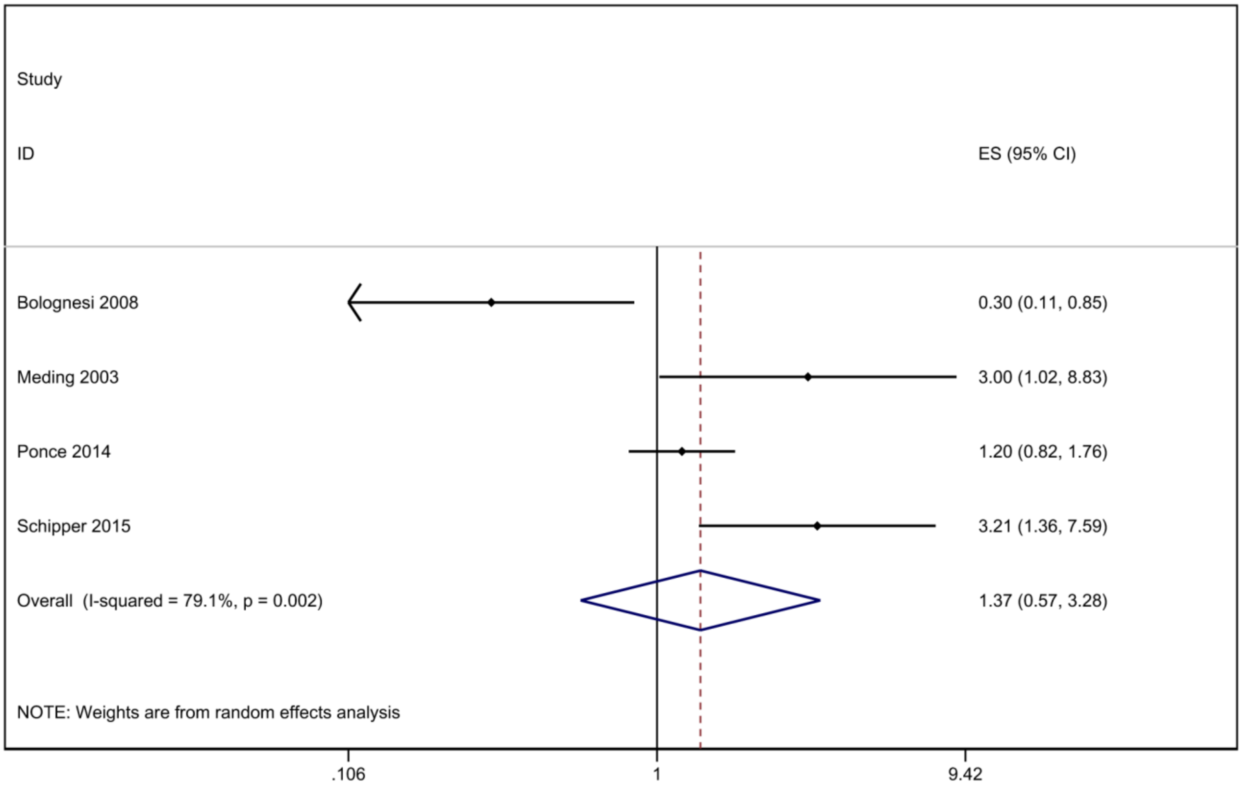

Figure S31. Forest plot of odds ratio of postoperative myocardial infarction in patients with DM vs those without DM in orthopedic surgery

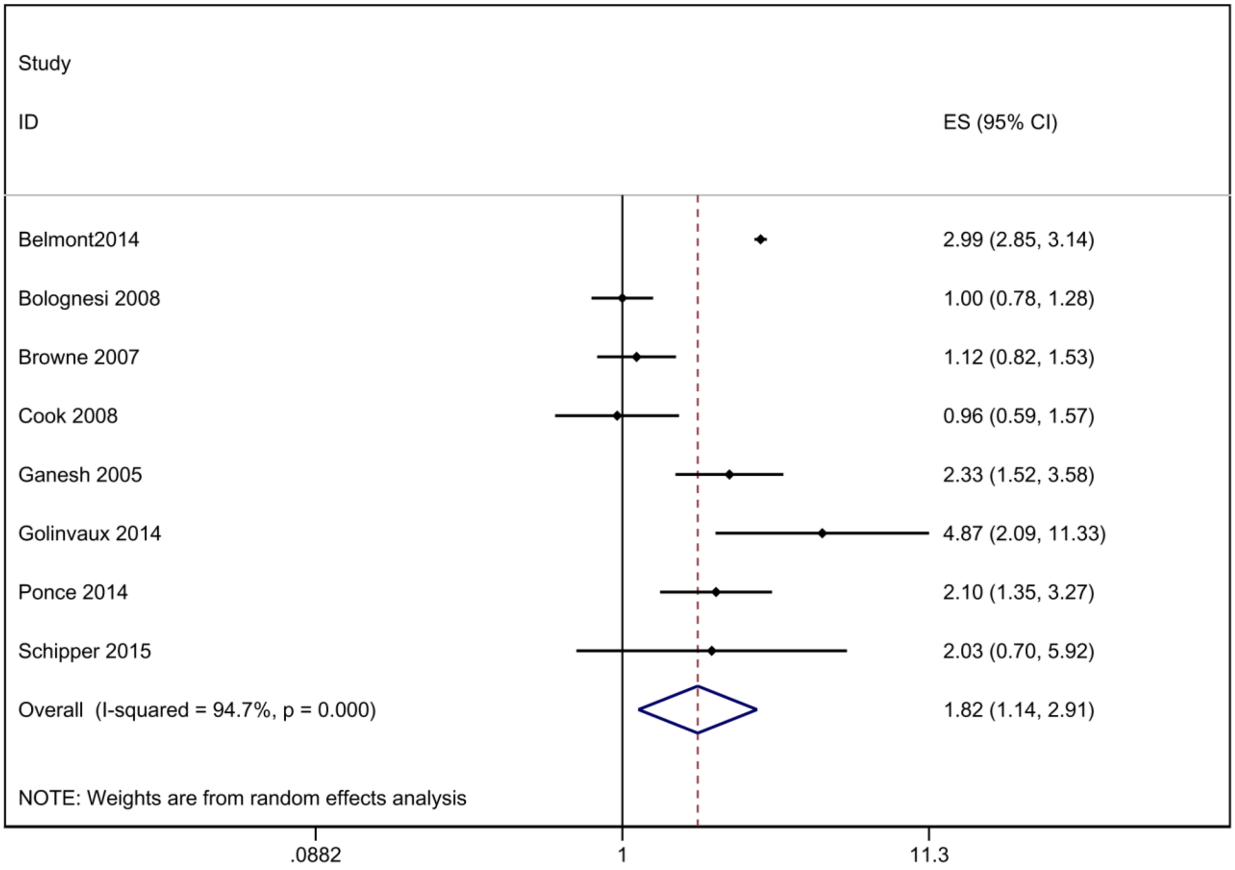

Figure S32. Forest plot of odds ratio of postoperative mortality in patients with DM vs those without DM in general surgery in orthopedic surgery

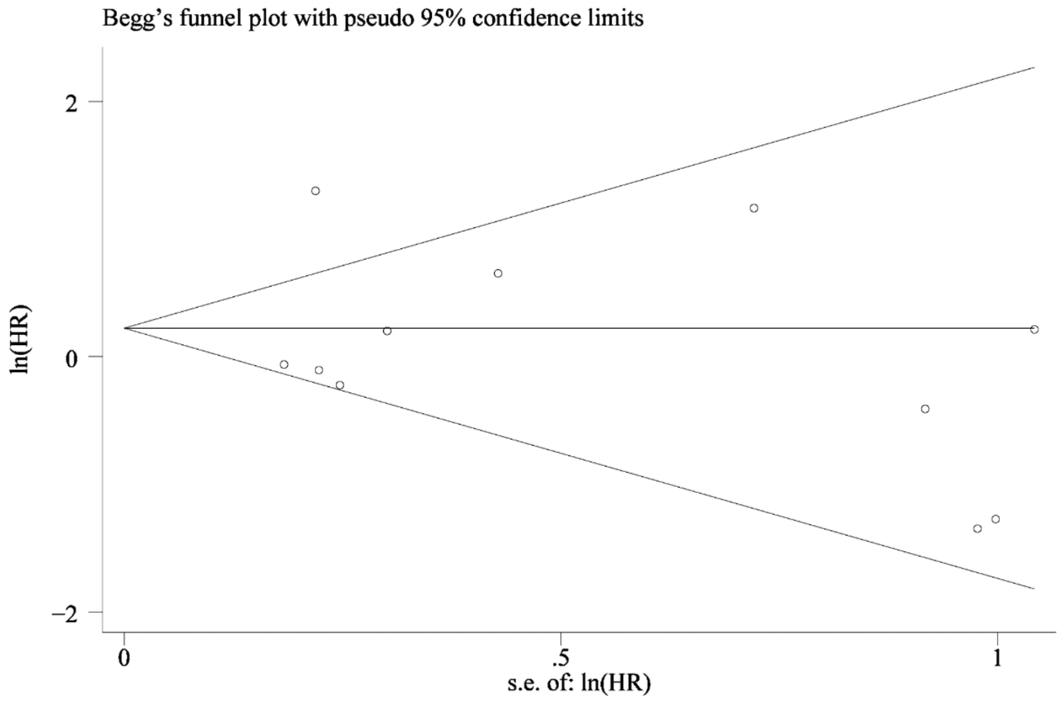

Figure S33. Funnel plot of odds ratio of postoperative venous thromboembolism in patients with DM vs those without DM

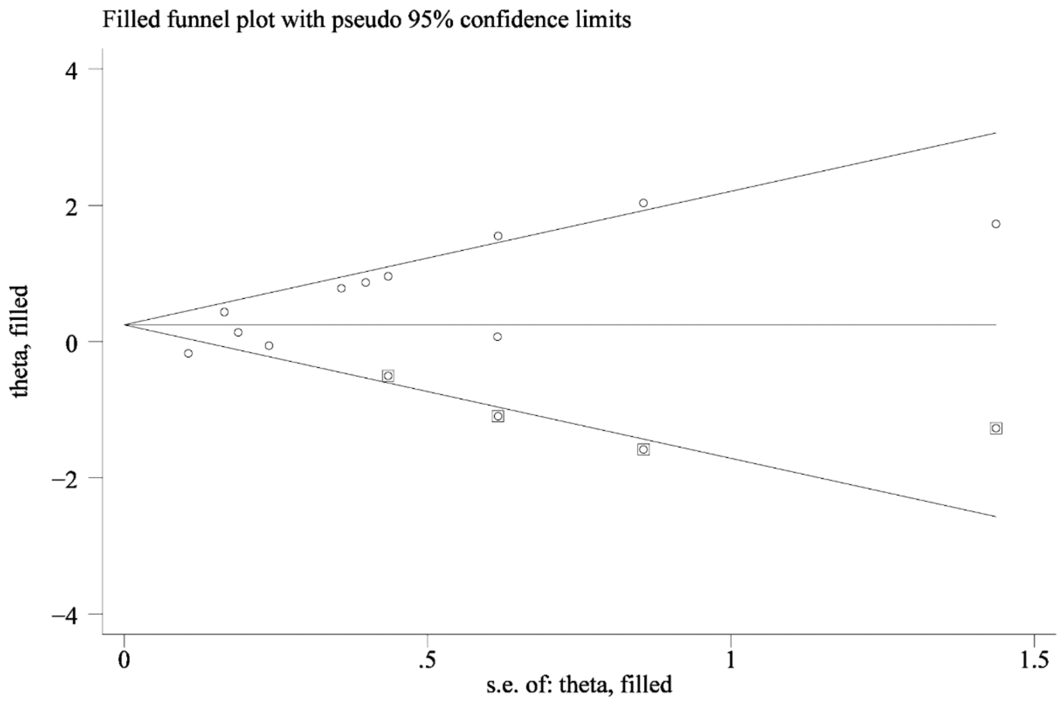

Figure S34. Funnel plot of odds ratio of reoperation in patients with DM vs those without DM

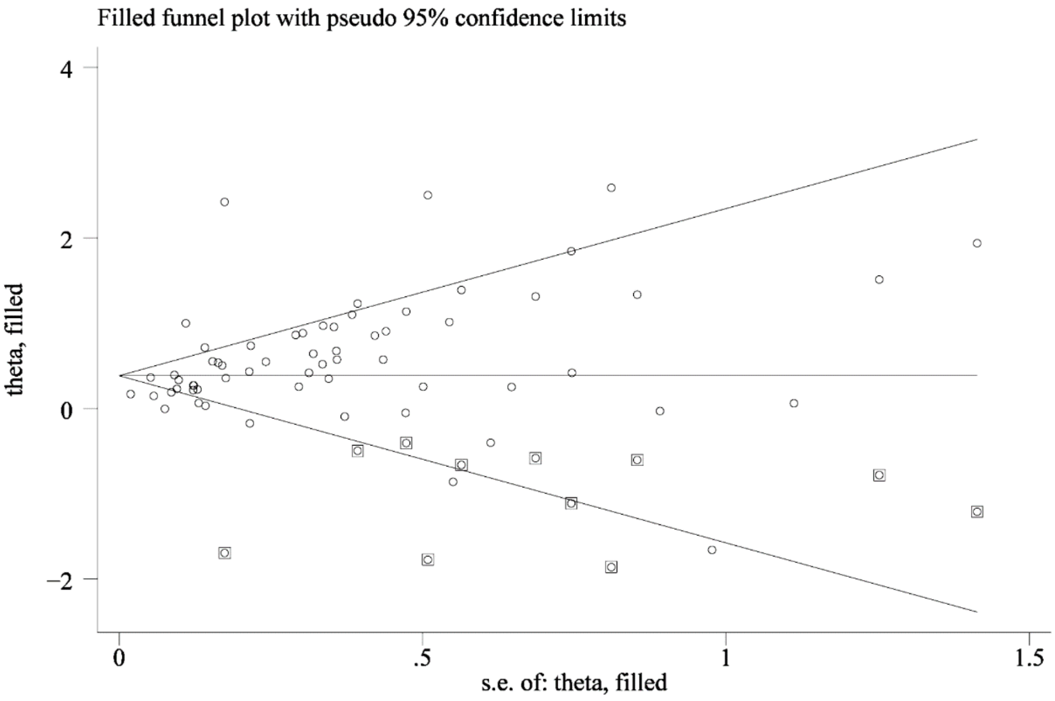

Figure S35. Funnel plot of odds ratio of any postoperative complication in patients with DM vs those without DM

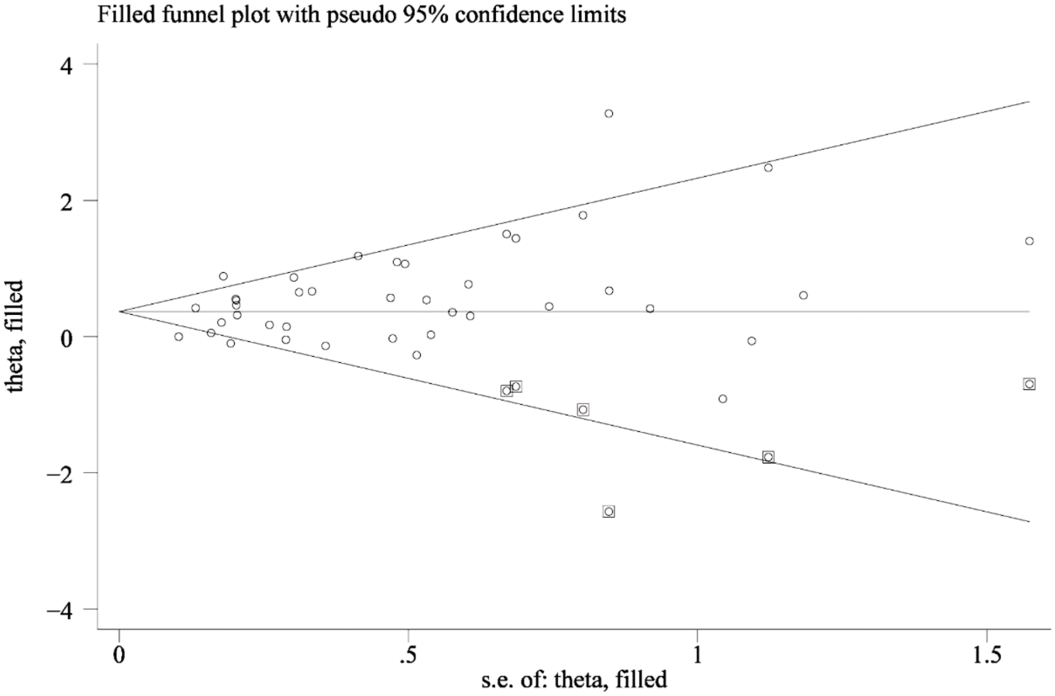

Figure S36. Funnel plot of odds ratio of postoperative infection in patients with DM vs those without DM

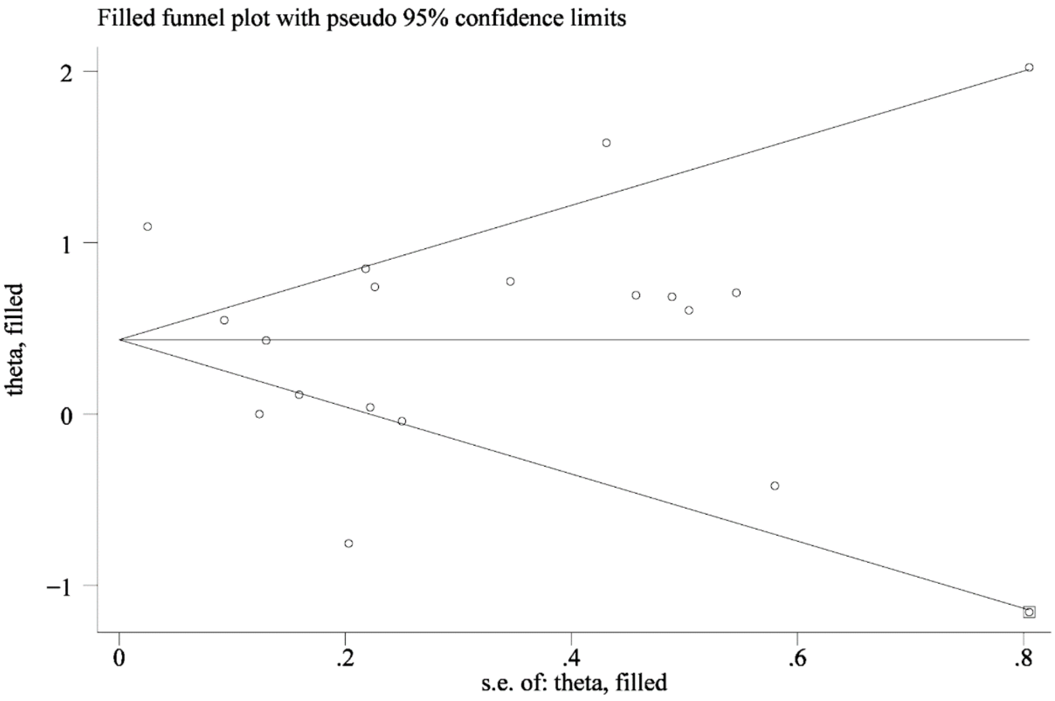

Figure S37. Funnel plot of odds ratio of readmission in patients with DM vs those without DM

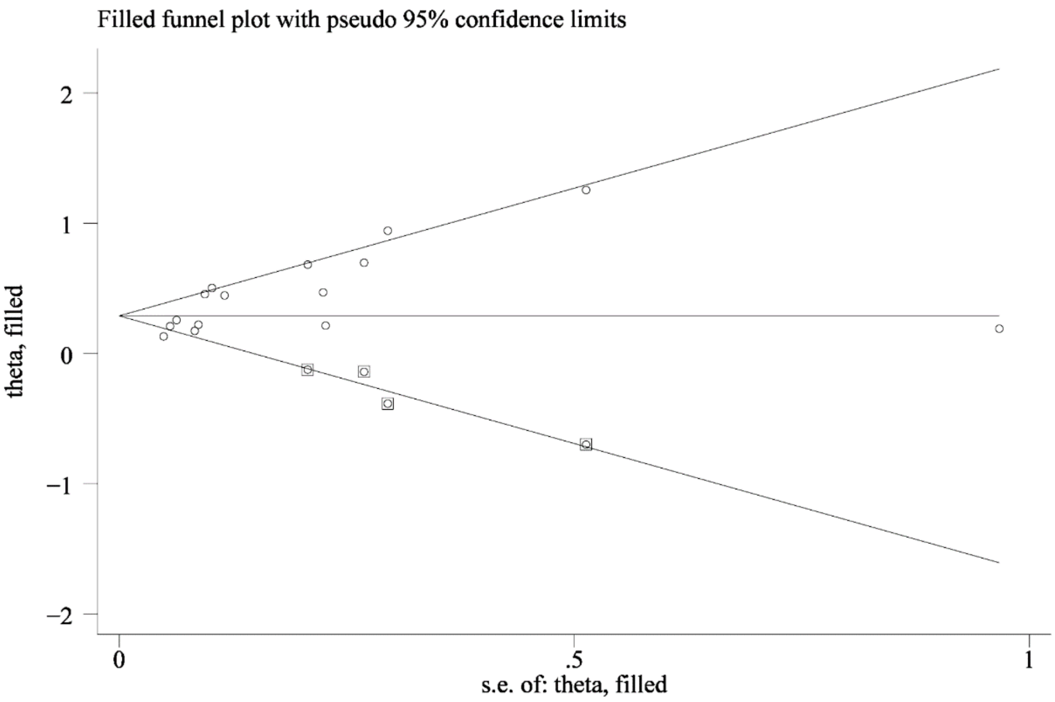

Figure S38. Funnel plot of odds ratio of postoperative mortality in patients with DM vs those without DM
